# Supplementary material for: Evaluation of the effect of reinforced education on the satisfaction of patients undergoing colonoscopy: A randomized controlled trial
Source: PLoS One. 2024 Jan 5;19(1):e0296126. doi: 10.1371/journal.pone.0296126 (PMC10769074; doi:10.1371/journal.pone.0296126)
Supplement: S1 Protocol — (DOCX) [file pone.0296126.s001.docx]

***STUDY PROTOCOL***

**Research Method**

The current study is a semi-experimental study in which the effect of the independent variable (use of media in patient education) on the dependent variable (satisfaction of patients undergoing colonoscopy) will be investigated.

**Type of Research**

The current research is semi-experimental and includes the implementation of media intervention in the education of colonoscopy patients referred to Ayatollah Taleghani Medical Education Center.

**Research Sample**

The statistical population of the present study will include colonoscopy patients referred to Ayatollah Taleghani Medical Education Center and the research sample will be patients with inclusion criteria.

**Adult sample size**

The number of samples is obtained using the following relationship in each group:

$$n\geq2\frac{\left( z_{\alpha/2}+z_{\beta} \right)^{2} \sigma^{2}}{(\mu_{1}- \mu_{2})}$$

where the probability of type 1 error:

$$\alpha=0.05 \to z_{\alpha/2}=1.96$$

Probability of type II error:

$$\beta=0.10 \to z_{\beta}=1.28$$

$$1- \beta=0.90$$

Power of observed effect size:

$$\left( \mu_{1}- \mu_{2} \right)/{\sigma=0.75}$$

${\mu_{2},\mu}_{1}$indicate the average satisfaction score of the adults.

Minimum sample required:

$$n=2\left( 1.96+1.28 \right)^{2} \left( \frac{1}{0.75} \right)^{2} =37$$

At least 37 adults in each group should be considered for the research, and considering 40% attrition, the number of samples is 50 patients in each group.

**Sampling**

In this research, sampling will be done by available method and the participants will be selected purposefully in two control and intervention groups. The similarity of the two groups in terms of age, sex, education level, disease, background and cause of hospitalization will be statistically investigated.

**Inclusion criteria**

1. Patients who do not have movement disorder.

2. Patients who do not have hearing or vision impairment.

3. Patients who do not have a medical history of suffering from cognitive diseases and depression.

4. Patients who do not take neuroleptics.

5. Patients who have not recently experienced a new crisis such as the death of loved ones, hospitalization, etc.

6. Have access to virtual space and educational videos.

**Exclusion criteria**

Patients who experience a new crisis during the research.

**Research environment**

The research environment is the Colonoscopy Department of Ayatollah Taleghani Medical Education Center.

**Research tools:**

The data collection tool in this research is as follows:

1- The demographic information questionnaire has 20 questions including the patient's age, sex, education, marital status, occupation, type of health insurance, reason for hospitalization, duration of hospitalization, history of illness, history of underlying illness, history of illness in the family (Appendix No. 1).

2- Colonoscopy satisfaction questionnaire (Appendix No. 2).

**Validity and reliability of tools**

In this research, to determine the validity of the used questionnaires, the method of determining face and content validity will be used (27, 28). The above questionnaires are a combination of different studies. Therefore, its validity and reliability will be checked. The validity check will be done by the method of form and content validity check, and in the reliability check based on the type of instrument, the Cronbach's alpha method and the test-retest will be used. In order to check face validity and qualitative content validity, the tools will be available to 10 faculty members of Shahid Beheshti University of Medical Sciences and experts and nurses with experience in the colonoscopy department, and after collecting their opinions, the necessary corrections will be made. It will be done in tools.

In the next step, content validity will be checked in a quantitative way, using the opinions of 10 members of the Faculty of Nursing and Midwifery of Shahid Beheshti University of Medical Sciences and experts and nurses with experience in colonoscopy regarding simplicity, ambiguity, the relevance of the items, the validity index of the content and the necessity of the items are calculated. The content validity index is higher than 0.7 in each case of simplicity, ambiguity, relevance in each item. The content validity coefficient based on the number of professors participating in the validity review (minimum 10 people are considered) is 0.49 acceptable according to the Lavshe table (27,28)^[[1]](#footnote-1)^. Cronbach's alpha and test-retest will be used for the reliability of the tool (27, 28).

**Data collection method**

In the present study, data will be collected by completing a questionnaire. In this way, after obtaining the code of ethics from the ethics committee and the letter of introduction from Shahid Beheshti University of Medical Sciences and Health Services, the researcher explains the goals and working methods to them through coordination with the officials of the Ayatollah Taleghani Medical Education Center and will conduct the study.

In order to collect data from the patients, first a written consent to participate in the research will be obtained from them, after explaining the objectives and the method of doing the work to participate in the research. Both groups complete the demographic questionnaire (Appendix 1) and the colonoscopy satisfaction questionnaire (Appendix No. 2) (colonoscopy) and the results are compared in the two control and intervention groups.

**data analysis method**

Analyzing the research data using statistical package software for social sciences version 20, and using descriptive tests such as mean, variance, and standard deviation, and using inferential tests such as chi-square, independent t-test, and analysis of variance, the data will be analyzed. The Smirnov Kolmograph test will be used to check the normality of the data. To compare the two groups, chi-square, Menio-Whitney and independent t-tests will be used, and to compare the results in the intervention and control groups, independent t-test or Menio-Whitney statistical tests will be used. In all tests, a significance level of less than 0.05 will be considered.

**Attachments**

***Appendix No. 1:***

***Questionnaire of demographic information***

1- Age: ............. years

2- Gender: male or female

3- Marital status: single, married, divorced, widowed

4- Education:...............

5- Occupation:............

6- Reason for admission:............

7- Hospitalization period:............

8- Type of health insurance:..............

9- Amount of income:..............

10- History of drug use: yes, no

11- History of cancer in other family members: yes, no

12- History of other underlying diseases: yes, no

***Appendix No. 2:***

***Colonoscopy satisfaction questionnaire***

**Before colonoscopy**

1) Have you been seen as an outpatient by a doctor to discuss your condition and the planned catheterization procedure?

Yes, No

2) Has the doctor explained what is done during the colonoscopy procedure?

Absolutely, To Some Extent, No

3) During the pre-op clinic visit, were you told what to do after the colonoscopy?

Absolutely, To Some Extent, No

4) Have you been given enough information before the colonoscopy procedure?

Yes, No

5) Has the doctor or nurse explained what will be done during the procedure during the visit before the colonoscopy?

Absolutely, To Some Extent, No

6) Did you have unanswered questions about the procedure before being admitted to the hospital?

Yes, No

**During colonoscopy**

7) If you were visited by a doctor in an outpatient clinic, how would you evaluate his visit?

Weak, good, not good, average, good, excellent, not usable

8) Did you have a chance to ask the remaining questions?

Yes, No

9) Did you feel ready for the operation?

Yes, No

10) Was your experience in the colonoscopy unit what you expected?

Yes, No

**After colonoscopy**

11) After the operation, did one of the colonoscopy unit staff explain what happened?

Yes, No

12) When you left the colonoscopy unit, were you aware of the results?

Yes, No

13) Did you feel ready to be discharged when you left the hospital?

Yes, No

14) How satisfied were you with your experience when you left the hospital?

Not At All, Low, Moderate, Completely Satisfied, Very Satisfied

**Discharge time from the hospital**

15) Did you have any complications as a result of your colonoscopy?

Yes, No

16) Would you recommend this hospital to others for colonoscopy?

Yes, No

1. :

   27. Polit DF, Yang F. Measurement and the measurement of change: a primer for the health professions: Wolters Kluwer Health; 2015.

   28. Kellar SP, Kelvin EA. Munro's statistical methods for health care research: Wolters Kluwer Health/Lippincott Williams & Wilkins; 2013. [↑](#footnote-ref-1)
