# Supplementary material for: Evaluation of the effect of reinforced education on the satisfaction of patients undergoing colonoscopy: A randomized controlled trial
Source: PLoS One. 2024 Jan 5;19(1):e0296126. doi: 10.1371/journal.pone.0296126 (PMC10769074; doi:10.1371/journal.pone.0296126)
Supplement: Attachment — Submitted filename: PONE-D-23-02398_R1 (1).pdf [file pone.0296126.s006.pdf]

PLOS ONE

Evaluation of the effect of reinforced education on the satisfaction of patients undergoing colonoscopy: a randomized controlled trial

--Manuscript Draft--

**Manuscript Number:**	PONE-D-23-02398R1
**Article Type:**	Clinical Trial
**Full Title:**	Evaluation of the effect of reinforced education on the satisfaction of patients undergoing colonoscopy: a randomized controlled trial
**Short Title:**	Effect of Reinforced Education on the Satisfaction of Patients Undergoing Colonoscopy
**Corresponding Author:**	Negin - Farid, Nursing undergraduate student<br>Shahid Beheshti University of Medical Sciences School of Nursing and Midwifery<br>Tehran, IRAN, ISLAMIC REPUBLIC OF
**Keywords:**	colonoscopy; satisfaction; bowel preparation; reinforced education
**Abstract:**	Introduction<br>Fourteen million colonoscopies are performed annually in the United States, where the colonoscopy results depend on bowel preparation before the procedure. However, suitable preparation is not performed in 20-25% of cases, resulting in increased loss of time, failure to diagnose, procedure repetition, and decreased patient satisfaction. Therefore, this study was performed to evaluate the effect of reinforced education (RE) on the satisfaction of patients undergoing colonoscopy.<br>Methods<br>The current study applied an experimental research method. Participants in the study included 83 patients undergoing colonoscopy referred to Ayatollah Taleghani Medical Educational Center, divided into control and intervention groups. In the control group, standard education was administered, whereas in the intervention group, the patient was instructed using media and virtually, and preparation was followed up the day before the colonoscopy. The research tool was a demographic and satisfaction questionnaire developed by the researcher. Analyses were conducted using IBM SPSS software (v. 20).<br>Results<br>Eighty-three patients, including 47 men and 36 women with an average age of 49.19 years, participated in the study. Mean (standard deviation) patient satisfaction was 11.78 (4.65) in the intervention group and 9.04 (2.95) in the control group; the independent t-test revealed a significant difference between the two groups (P<0.001).<br>Conclusions<br>The study suggests that reinforced education using media and virtual means is effective in the satisfaction of patients undergoing colonoscopy.
**Order of Authors:**	Negin - Farid, Nursing undergraduate student
	Shakila - Sharifian, Nursing undergraduate student
	Raziyeh - Ghafouri, PhD in Nursing, Assistant professor,
**Response to Reviewers:**	Thank you for giving us the opportunity to submit a revised draft of our manuscript titled [Evaluation of the effect of reinforced education on the satisfaction of the patients undergoing colonoscopy: a randomized controlled trial] to PLOS ONE. We appreciate the time and effort that you and the reviewers have dedicated to providing your valuable feedback in our manuscript. We are grateful to the reviewers for their insightful comments on our paper. We have been able to incorporate changes to reflect most of the suggestions provided by the reviewers.We have highlighted the changes within the manuscript.<br><br>I will thank you in advance to see the track change file and also the Response to Reviewer.
**Additional Information:**	
**Question**	**Response**

**Financial Disclosure**<br><br>Enter a financial disclosure statement that describes the sources of funding for the work included in this submission. Review the submission guidelines for detailed requirements. View published research articles from *PLOS ONE* for specific examples.<br><br>This statement is required for submission and **will appear in the published article** if the submission is accepted. Please make sure it is accurate.<br><br>**Unfunded studies**<br>Enter: *The author(s) received no specific funding for this work.*<br><br>**Funded studies**<br>Enter a statement with the following details:<br>• Initials of the authors who received each award<br>• Grant numbers awarded to each author<br>• The full name of each funder<br>• URL of each funder website<br>• Did the sponsors or funders play any role in the study design, data collection and analysis, decision to publish, or preparation of the manuscript?<br>• **NO** - Include this sentence at the end of your statement: *The funders had no role in study design, data collection and analysis, decision to publish, or preparation of the manuscript.*<br>• **YES** - Specify the role(s) played.<br><br>* typeset	The author(s) received no specific funding for this work.
**Competing Interests**<br><br>Use the instructions below to enter a competing interest statement for this submission. On behalf of all authors, disclose any competing interests that could be perceived to bias this work—acknowledging all financial support and any other relevant financial or non-financial competing interests.<br><br>This statement is required for submission	The authors have declared that no competing interests exist.

Powered by Editorial Manager® and ProduXion Manager® from Aries Systems Corporation

and **will appear in the published article** if the submission is accepted. Please make sure it is accurate and that any funding sources listed in your Funding Information later in the submission form are also declared in your Financial Disclosure statement.<br><br>View published research articles from *PLOS ONE* for specific examples.<br><br>**NO authors have competing interests**<br><br>Enter: *The authors have declared that no competing interests exist.*<br><br>**Authors with competing interests**<br><br>Enter competing interest details beginning with this statement:<br><br>*I have read the journal's policy and the authors of this manuscript have the following competing interests: [insert competing interests here]*<br><br>* typeset	
**Ethics Statement**<br><br>Enter an ethics statement for this submission. This statement is required if the study involved:<br><br>• Human participants<br>• Human specimens or tissue<br>• Vertebrate animals or cephalopods<br>• Vertebrate embryos or tissues<br>• Field research<br><br>Write "N/A" if the submission does not require an ethics statement.<br><br>General guidance is provided below. Consult the submission guidelines for detailed instructions. **Make sure that all information entered here is included in the Methods section of the manuscript.**	The study protocol was approved with the Ethics Committee of Student Research Committee at Shahid Beheshti University of Medical Sciences (Ethics Approval No: IR.SBMU.RETECH.REC. 1400.1082). To observe the ethical considerations, the research goals and procedures were elucidated to the participants, they were assured of information anonymity and confidentiality, and informed written consent was obtained from each participants. They participated in the study voluntarily and could leave the study at any stage.

**Format for specific study types**<br><br>**Human Subject Research (involving human participants and/or tissue)**<br>• Give the name of the institutional review board or ethics committee that approved the study<br>• Include the approval number and/or a statement indicating approval of this research<br>• Indicate the form of consent obtained (written/oral) or the reason that consent was not obtained (e.g. the data were analyzed anonymously)<br><br>**Animal Research (involving vertebrate animals, embryos or tissues)**<br>• Provide the name of the Institutional Animal Care and Use Committee (IACUC) or other relevant ethics board that reviewed the study protocol, and indicate whether they approved this research or granted a formal waiver of ethical approval<br>• Include an approval number if one was obtained<br>• If the study involved *non-human primates*, add *additional details* about animal welfare and steps taken to ameliorate suffering<br>• If anesthesia, euthanasia, or any kind of animal sacrifice is part of the study, include briefly which substances and/or methods were applied<br><br>**Field Research**<br><br>Include the following details if this study involves the collection of plant, animal, or other materials from a natural setting:<br>• Field permit number<br>• Name of the institution or relevant body that granted permission	
**Data Availability**<br><br>Authors are required to make all data underlying the findings described fully available, without restriction, and from the time of publication. PLOS allows rare exceptions to address legal and ethical concerns. See the PLOS Data Policy and FAQ for detailed information.	Yes - all data are fully available without restriction

A Data Availability Statement describing where the data can be found is required at submission. Your answers to this question constitute the Data Availability Statement and **will be published in the article**, if accepted.<br><br>**Important:** Stating 'data available on request from the author' is not sufficient. If your data are only available upon request, select 'No' for the first question and explain your exceptional situation in the text box.<br><br>Do the authors confirm that all data underlying the findings described in their manuscript are fully available without restriction?	
**Describe where the data may be found in full sentences. If you are copying our sample text, replace any instances of XXX with the appropriate details.**<br><br>• If the data are **held or will be held in a public repository**, include URLs, accession numbers or DOIs. If this information will only be available after acceptance, indicate this by ticking the box below. For example: *All XXX files are available from the XXX database (accession number(s) XXX, XXX.)*.<br>• If the data are all contained **within the manuscript and/or Supporting Information files**, enter the following: *All relevant data are within the manuscript and its Supporting Information files.*<br>• If neither of these applies but you are able to provide **details of access elsewhere**, with or without limitations, please do so. For example:<br><br>*Data cannot be shared publicly because of [XXX]. Data are available from the XXX Institutional Data Access / Ethics Committee (contact via XXX) for researchers who meet the criteria for access to confidential data.*<br><br>*The data underlying the results presented in the study are available from (include the name of the third party*	All relevant data are within the manuscript and its Supporting Information files.

and contact information or URL).<br>• This text is appropriate if the data are owned by a third party and authors do not have permission to share the data.<br><br>* typeset	
Additional data availability information:	

Manuscript Click here to access/download;Manuscript;Manuscript.docx

Evaluation of the effect of reinforced education on the satisfaction of patients undergoing colonoscopy: a randomized controlled trial

Negin Farid[1*], Shakila Sharifian[1], Raziyeh Ghafouri[2]

[1] Student Research Committee, School of Nursing & Midwifery, Shahid Beheshti University of Medical Sciences, Tehran, Iran

[2] Department of Medical and Surgical, School of Nursing & Midwifery, Shahid Beheshti University of Medical Sciences, Tehran, Iran

[*]Corresponding author:

E-mail: neginfarid.79@gmail.com, n.farid@sbmu.ac.ir (NF)

This study was registered using code IRCT ID IRCT20210131050189N5 in the Iranian Registry of Clinical Trials.

Full title: Evaluation of the effect of reinforced education on the satisfaction of patients undergoing colonoscopy: a randomized controlled trial

Short title: Effect of reinforced education on the satisfaction of patients undergoing colonoscopy

Abstract

Introduction

Fourteen million colonoscopies are performed annually in the United States, where the colonoscopy results depend on bowel preparation before the procedure. However, suitable preparation is not performed in 20-25% of cases, resulting in increased loss of time, failure to diagnose, procedure repetition, and decreased patient satisfaction. Therefore, this study was performed to evaluate the effect of reinforced education (RE) on the satisfaction of patients undergoing colonoscopy.

Methods

The current study applied an experimental research method. Participants in the study included 83 patients undergoing colonoscopy referred to Ayatollah Taleghani Medical Educational Center, divided into control and intervention groups. In the control group, standard education was administered, whereas in the intervention group, the patient was instructed using media and virtually, and preparation was followed up the day before the colonoscopy. The research tool was a demographic and satisfaction questionnaire developed by the researcher. Analyses were conducted using IBM SPSS software (v. 20).

Results

Eighty-three patients, including 47 men and 36 women with an average age of 49.19 years, participated in the study. Mean (standard deviation) patient satisfaction was 11.78 (4.65) in the intervention group and 9.04 (2.95) in the control group; the independent t-test revealed a significant difference between the two groups ($P<0.001$).

Conclusions

The study suggests that reinforced education using media and virtual means is effective in the satisfaction of patients undergoing colonoscopy.

Keywords*:* colonoscopy; satisfaction; bowel preparation; reinforced education

Introduction

Fourteen million colonoscopies are performed annually in the United States (1), where colonoscopy results depend on bowel preparation before the procedure (1, 2). Inadequate preparation fails to diagnose 42% of adenomas and 27% of advanced adenomas. Proper colon preparation facilitates a more thorough examination of the mucosa, the presence of polyps and adenomas, and early cancer diagnosis (1). In 20-25% of cases, however, adequate preparation is lacking (3), resulting in an increased loss of time, failure to diagnose, an increase in cost (3, 4), and a higher risk of procedural adverse events, such as bleeding or perforation (4).

Although proper bowel preparation is the most important factor in colonoscopy (3), patients frequently find it uncomfortable (5). One in five individuals fails to take the prescribed dose of colonoscopy preparation drugs (1). Gender, constipation, drug use, previous colon surgery, diabetes, obesity, neurological disease, and lack of knowledge influence inadequate preparation (3). Examining the patient's needs and educating them on the significance of colonoscopy preparation is one of the primary obstacles (3). Consequently, effective education can increase a patient's willingness to comply with instructions (1).

Education is provided through various methods, such as pamphlets, animations, messages, and videos, which increase patient readiness by 11-36% (2). If multiple instructional strategies are employed in education, they will be more effective (6). Furthermore, the training will be more influential if it is based on problem-solving techniques, the immediate need of the patient, and an appreciation of its significance (7).

Due to the significance of patient compliance and bowel preparation in colonoscopy procedures, training can be effective if it is patient-centered (8), interactive (5), individually focused (3) based on the patient's needs, and one that emphasizes the importance of bowel preparation (9). Patient acceptance is improved by all educational methods, including oral and

written education (3), face-to-face education, and media-based education (10). However, new methods such as utilizing technology, media (1), web applications (11), 3D online programs (12), and mobile applications (13, 14) may increase patient acceptance to a greater extent, necessitating additional research on their efficacy (1). Media use can make reinforced education (RE) more effective from admission until the colonoscopy day (15).

The level of patient satisfaction is a crucial criterion for evaluating the quality of service (16). Patient satisfaction is an indicator of the quality of care, which is a vital consideration when selecting a treatment center; therefore, it is essential to conduct a study to determine how to improve the quality of services and patient satisfaction (17). More research must be conducted to boost care recipients' satisfaction (18, 19). Patient dissatisfaction is caused by insufficient preparation, incorrect diagnosis, and repeated colonoscopies, which can be remedied through proper training and follow-up. Based on the information above, it can be concluded that patient satisfaction and bowel preparation can be improved using RE. Therefore, this study evaluated the effect of RE on the satisfaction of colonoscopy patients.

Methods

This semi-experimental study evaluated the impact of using media and virtual means to educate Ayatollah Taleghani Medical Educational Center-referred colonoscopy patients. This medical center performs approximately 2,500 colonoscopies annually, or nearly 200 per month. The study's statistical population consisted of colonoscopy patients referred to the Ayatollah Taleghani Educational Medical Center, where the study sample comprised patients who met the inclusion criteria.

The study was registered with the Iranian Clinical Trial Registry (IRCT20210131050189N5). The authors confirm that all ongoing and related trials for this intervention are registered.

It should be mentioned that due to the type of intervention of this study, we were only satisfied with obtaining the code of ethics at the beginning. But after taking action to submit the article, and with a more detailed study of the WHO guidelines, we realized the necessity of registering our protocol in the trials registration center. Therefore, retrospectively and by obtaining the necessary permits, the trial was successfully registered.

Sample size/power:

The number of samples in each group was determined by the following equation:

$$n \geq 2\frac{\left(z_{\alpha/2} + z_{\beta}\right)^2 \sigma^2}{(\mu_1 - \mu_2)}$$

Probability of type 1 error: $\alpha = 0.05 \rightarrow z_{\alpha/2} = 1.96$

Probability of type 2 error: $\beta = 0.10 \rightarrow z_{\beta} = 1.28$

Power of observed effect size: $(\mu_1 - \mu_2)/\sigma = 0.75$

Minimum required sample: $n = 2(1.96 + 1.28)^2 \left(\frac{1}{0.75}\right)^2 = 37$

Each group required a minimum of 37 participants. Considering a potential 40% decline in population, the sample size for each group consisted of 50 patients.

Data collection and sampling:

The sampling period lasted from May 22 to June 22. Data collection was completed through August 7. The inclusion criteria were the absence of movement, hearing, and vision disorders,

a medical history free of cognitive diseases and depression, the non-use of neuroleptics, the absence of a recent tragedy such as the death of a loved one or hospitalization, and access to online resources and educational videos. Patients who experienced a new tragedy during the research or refused to continue cooperating were excluded.

Red and blue colored cards were used to divide the participants into the control and intervention groups (Fig 1). *RG* designed the allocation sequence, *NF* and *SS* enrolled participants, and *NF* and *RG* assigned interventions to each group (*capital letters indicate the authors' initials*). Also patients in each group were blinded to their group classification.

The two groups were homogeneous, where no statistically significant differences were observed in age, sex, education level, underlying disease, or reason for admission ($P<0.05$).

Fig 1. CONSORT flowchart for the study

Data collection tool:

[illegible] data collection tool included a demographic information questionnaire with 20 questions, including the patient's age, sex, level of education, marital status, occupation, type of health insurance, the reason for hospitalization, length of hospitalization, illness history, underlying disease, medical family history, previous colonoscopy experience, place of residence, diet (daily fruit consumption, weekly fast food consumption), ability to perform daily activities, and amount of exercise per day.

The colonoscopy satisfaction instrument was a researcher-made instrument with 12 questions in 3 dimensions (pre-colonoscopy education, colonoscopy experience, and colonoscopy result). Before preparing the tool, its components were developed based on a review of the relevant literature (20-23). Then, duplicate items were eliminated, categorized, and summarized by the expert panel consisting of ten gastroenterologists and endoscopy

nursing experts. Next, face validity (qualitative), content validity (quantitative and qualitative), and construct validity were examined. Ten nurses were asked to provide their corrections regarding the use of correct grammar, vocabulary, placement of expressions, and provision of sufficient points to verify the accuracy of the content. The content validity index (CVI) and content validity ratio (CVR) were then determined and validated. The relationship between the items was then determined and summarized using exploratory factor analysis into three factors (pre-colonoscopy education, colonoscopy experience, and colonoscopy result). Cronbach's alpha was used to test reliability. Cronbach's alpha of the instrument was 0.83.

The colonoscopy patients were contacted one week after the procedure, and the aforementioned patient satisfaction questionnaire was administered.

[illegible]tervention:

The standard training program was provided to the control group, which received a printed training sheet. In the intervention group, patients were contacted the day before their colonoscopy, and training materials, including video clips, an application, and an Instagram page, were sent to them via the WhatsApp application, and their questions were answered.

Bisacodyl tablets and Pidrolax powder were utilized for Bowel preparation. Below are the morning and afternoon versions of the routine protocol for bowel preparation (as recommended by ESGE (24)).

Colonoscopy appointment between 8:00 to 13:00

One day before a colonoscopy, only liquids, including water, sugar water, compote water, tea, strained fruit juice (clear and pulp-free), meat broth, and chicken broth, may be consumed beginning at 9 am. Avoid soups, dairy products, juices containing fruit particles, compotes, and solid foods such as (bread, rice, potatoes, meat, and ash).

Maintain a fluid-only diet throughout the day. Use one tablet of Bisacodyl at noon, 4 pm, and 8 pm.

Use six colonoscopy preparation powders (Pidrolox) between noon and midnight. Each powder is mixed with one liter of water (four large glasses), and one glass is consumed every half-hour.

At least three to four hours of walking and physical activity are required daily.

Start fasting in the morning on the day of the test.

Colonoscopy appointment between 13:00 to 19:00

One day before the colonoscopy, only liquids, including water, sugar water, compote water, tea, strained fruit juice (clear and pulp-free), meat broth, and chicken broth, may be consumed beginning at 9:00 am. Avoid soups, dairy products, fruit juices containing fruit particles, compotes, and solid foods such as (bread, rice, potatoes, meat, and ash).

Maintain a fluid-only diet throughout the day. Use one tablet of Bisacodyl at noon, 4:00, and 8:00 pm.

Use five powders for colonoscopy preparation between 4:00 pm and 12:00 am. Pour each powder into one liter of water (four large glasses), and consume one glass every 20 minutes.

At least three to four hours of walking and physical activity are required daily.

At 8:00 am on the colonoscopy day, a preparation powder should be mixed with one liter of water (four large glasses) and consumed. Fasting begins at 10 am.

Data analysis:

IBM SPSS software was employed for analyses (v. 20, IBM Corp., Armonk, NY). Kolmogorov-Smirnov tests were used to assess the normality of the distribution of variables. A p-value < 0.05 was deemed statistically significant. Either the t-test or the chi-square test was utilized to analyze demographic data. If the data distribution was normal, the paired t-test

and student t-test were used to compare variables within and between groups. Notably, we used the 2010 version of the CONSORT reporting guidelines for this manuscript (25).

Results

In this study, 83 patients with an average age of 49.19 years participated, including 47 men and 36 women. Table 1 shows the demographic characteristics of the intervention group (43 individuals) and the control group (40 individuals).

Table 1. Participants' demographic characteristics.

		Group			
		Control		Intervention	
		Count	N %	Count	N %
Gender	Female	18	21.7%	18	21.7%
	Male	25	30.1%	22	26.5%
Marital Status	Single	7	8.6%	6	7.4%
	Marriage	36	44.4%	32	39.5%
Education	School	17	21.5%	15	19.0%
	High School	19	24.1%	16	20.3%
	Bachelor	2	2.5%	4	5.1%
	Upper	2	2.5%	4	5.1%
	First	29	34.9%	19	22.9%

Number Colonoscopy	Second	10	12.0%	10	12.0%
	More	4	4.8%	11	13.3%
Hospitalization	Outpatient	33	39.8%	35	42.2%
	Inpatient	10	12.0%	5	6.0%
Family History	No	33	40.2%	29	35.4%
	Yes	10	12.2%	10	12.2%

Initially, we examined the homogeneity of the two groups and observed no significant difference ($P>0.05$). Mean (standard deviation) patient satisfaction was 11.78 (4.65) in the intervention group and 9.04 (2.95) in the control group. The independent t-test revealed a statistically significant difference between the two groups ($P<0.001$). In the colonoscopy experience factor, the t-test revealed that the difference was not statistically significant ($P>0.05$). In the colonoscopy results ($P=0.05$); and pre-colonoscopy education, the t-test indicated that the difference was statistically significant ($P<0.001$). The results of patient satisfaction factors between the control and intervention groups are shown in Table 2.

Table 2. Comparative analysis of patient satisfaction between the control and intervention groups.

	Control		Intervention			
	Mean	Standard Deviation	Mean	Standard Deviation	T	P value
pre-colonoscopy education	1.66	1.68	3.55	2.51	-3.99	0.00

colonoscopy experience	4.57	2.17	4.95	2.13	-0.68	0.49
colonoscopy result	1.30	0.66	1.63	0.77	-1.99	0.05

Discussion

The present study aimed to evaluate the effect of RE on the level of satisfaction of patients undergoing colonoscopy. The results indicated that RE using media and virtual means improved the level of satisfaction of these patients (P<0.01). Guo et al. observed similar effects of RE on bowl preparation (9). In this regard, Aggarwal et al. have studied the effect of training on safety, stated that effective training methods should be utilized concerning patient safety, and emphasized the importance of continuing training (26). Bott et al. compared the impact of implementing a specialized training program after treating cardiac arrhythmia to standard discharge instructions in their study. According to the study's findings, the training provided by the nurse to the patient 24 to 72 hours after discharge increased patient satisfaction and reduced readmission rates (27). The results of Bott et al. are consistent with the current study.

In this regard, Lutjeboer et al. demonstrated that promoting self-care can affect and increase patient satisfaction. Their study was conducted in the radiology department (28). In the present study, patient-centered training increased patient satisfaction. Similarly, Ezzat et al. found that reducing complications during cardiac catheterization can increase patient satisfaction (17). Since the reduction of complications is a component of self-care training, their results are also consistent with those of the present study and indicate a rise in satisfaction following the promotion of self-care with training and follow-up of care and preparations.

Patient education is crucial for promoting self-management and decreasing hospitalization and treatment costs (27). Therefore, three to four dollars are saved in costs for

every dollar spent on patient education. According to presented statistics, approximately 69 to 100 million dollars are spent annually in the United States on medical issues resulting from a lack of education (28).

The majority of individuals possess cell phones today (29). Athilingam stated in 2016 that there are 7 billion cell phones worldwide and that nearly 95% of the global population uses cell phones (30). The owner's cell phone is with them throughout the day (30), and its use is beneficial for teaching self-care and disease prevention programs (31). Utilization and accessibility have increased satisfaction (32). The cell phone is one of the most widely used technological innovations that improve communication between the patient and the medical staff in the health system, and with suitable programs, higher attractiveness, and the possibility of remote patient follow-up, it has contributed to patient satisfaction (33, 34). The current study used mobile and media programs to improve patient education and follow-up. Mobile education in the reinforced educational program was more acceptable and increased patient satisfaction.

Even though our study group was limited to patients undergoing colonoscopy, and this study was conducted with random samples from an Iranian population, and should allude to future studies with much larger sample sizes collected at other geographical locations to confirm the efficacy of the RE procedure, it appears that the result can be generalized to other procedures where the background knowledge of the patient and their family is incomplete and limited, because it reduplicates the importance of the patient's and family's participation in the decision-making process.

Research Limitations

The outbreak of COVID-19, which hindered data collection, was one of the study's limitations; however, the use of virtual means and telephone follow-up of patients did not affect the findings. One of the strengths was the use of several teaching methods.

Conclusions

Based on the results, it can be concluded that through media and cyberspace utilization, RE is effective in the satisfaction of patients undergoing colonoscopy. It is important to note that the current findings are based solely on random samples derived from the Iranian population and that future studies with much larger sample sizes collected from other geographic regions are required to confirm the efficacy of the RE method.

List of abbreviations

RE: Reinforced Education

CVR: Content Validity Ratio

CVI: Content Validity Index

3D: Three Dimensions

Acknowledgments

We would like to thank all participants and those who assisted us in conducting the research, particularly the entire staff of the colonoscopy department at Ayatollah Taleghani Educational Medical Center and the Gastroenterology and Liver Diseases Research Center at the Research Institute for Gastroenterology and Liver Diseases of Shahid Beheshti University of Sciences.

Authors' contributions

NF and RG conceived and designed the study.

NF, SS, and RG developed methodology.

NF, SS, and RG gathered, analyzed, and interpreted the data.

NF and RG wrote and revised the manuscript.

RG supervised and coordinated all aspects of the work.

All authors have read and approved the manuscript.

Funding

We did not receive external funding for this research.

Consent for publication

Not applicable.

Availability of Data and Materials:

All data generated or analyzed during this study are included in this published article and its supplementary file.

Ethical Consideration

The Ethics Committee of the Student Research Committee at Shahid Beheshti University of Medical Sciences approved the study protocol (IR.SBMU.RETECH.REC. 1400.1082) entitled "Evaluation of the effect of reinforced education with media on patient education on colonoscopy satisfaction." To adhere to ethical considerations, the research objectives and methods were explained to the participants, they were assured of the anonymity and confidentiality of their information, and each participant provided written informed consent.

They participated voluntarily and had the option to withdraw at any time. This research was registered with IRCT ID IRCT20210131050189N5 in the Iranian Registry of Clinical Trials.

Competing interests

The authors declare that they have no competing interests and confirm that all ongoing and related trials for this intervention are registered.

References

1. Kurlander JE, Sondhi AR, Waljee AK, Menees SB, Connell CM, Schoenfeld PS, et al. How efficacious are patient education interventions to improve bowel preparation for colonoscopy? A systematic review. PloS one. 2016;11(10):e0164442.
2. Jeon SC, Kim JH, Kim SJ, Kwon HJ, Choi YJ, Jung K, et al. Effect of sending educational video clips via smartphone mobile messenger on bowel preparation before colonoscopy. Clinical endoscopy. 2019;52(1):53.
3. Elvas L, Brito D, Areia M, Carvalho R, Alves S, Saraiva S, et al. Impact of personalised patient education on bowel preparation for colonoscopy: prospective randomised controlled trial. GE-Portuguese Journal of Gastroenterology. 2017;24(1):22-30.
4. Sadeghi A, Rajabnia M, Bagheri M, Jamshidizadeh S, Saberi S, Shahnazi P, et al. Predictive factors of inadequate bowel preparation for elective colonoscopy. Gastroenterology and Hepatology From Bed to Bench. 2022;15(1):66.
5. Schooley B, San Nicolas-Rocca T, Burkhard R. Cloud-based multi-media systems for patient education and adherence: a pilot study to explore patient compliance with colonoscopy procedure preparation. Health Systems. 2021;10(2):89-103.

6. Marble N, Loescher LJ, Lim KH, Hiscox H. Use of technology for educating melanoma patients. Journal of Cancer Education. 2010;25(3):445-50.

7. Cook DJ, Moradkhani A, Douglas KSV, Prinsen SK, Fischer EN, Schroeder DR. Patient education self-management during surgical recovery: combining mobile (iPad) and a content management system. Telemedicine and e-Health. 2014;20(4):312-7.

8. Bernstein MT, Kong J, Sriranjan V, Reisdorf S, Restall G, Walker JR, et al. Evaluating information quality of revised patient education information on colonoscopy: It is new but is it improved? Interactive journal of medical research. 2019;8(1):e11938.

9. Guo X, Li X, Wang Z, Zhai J, Liu Q, Ding K, et al. Reinforced education improves the quality of bowel preparation for colonoscopy: An updated meta-analysis of randomized controlled trials. PloS one. 2020;15(4):e0231888.

10. Bernstein MT, Garber J, Faucher P, Reynolds KA, Restall G, Walker JR, et al. New Patient Education Video on Colonoscopy Preparation: Development and Evaluation Study. JMIR human factors. 2020;7(4):e15353.

11. Trasolini R, Nap-Hill E, Suzuki M, Galorport C, Yonge J, Amar J, et al. Internet-based patient education prior to colonoscopy: Prospective, observational study of a single center’s implementation, with objective assessment of bowel preparation quality and patient satisfaction. Journal of the Canadian Association of Gastroenterology. 2020;3(6):274-8.

12. Zhao Y, Xie F, Bai X, Yang A, Wu D. Educational virtual reality videos in improving bowel preparation quality and satisfaction of outpatients undergoing colonoscopy: protocol of a randomised controlled trial. BMJ open. 2019;9(8):e029483.

13. Walter B, Schmid R, von Delius S. A smartphone app for improvement of colonoscopy preparation (ColoprAPP): development and feasibility study. JMIR mHealth and uHealth. 2017;5(9):e7703.

14. Cho J, Lee S, Shin JA, Kim JH, Lee HS. The impact of patient education with a smartphone application on the quality of bowel preparation for screening colonoscopy. Clinical Endoscopy. 2017;50(5):479-85.

15. Walter B, Klare P, Strehle K, Aschenbeck J, Ludwig L, Dikopoulos N, et al. Improving the quality and acceptance of colonoscopy preparation by reinforced patient education with short message service: results from a randomized, multicenter study (PERICLES-II). Gastrointestinal endoscopy. 2019;89(3):506-13. e4.

16. Ghazi K, Foroughan M, Hosseini M, Hosseinzadeh S, Askari M. The client satisfaction of delivered services in private nursing homes for elderly: a survey in the provinces of Golestan, Mazandaran, Semnan and Northern Khorasan in 2012 or 1391. Journal of Sabzevar University of Medical Sciences. 2013;20(3):320-30.

17. Ezzat VA, Chew A, McCready JW, Lambiase PD, Chow AW, Lowe MD, et al. Catheter ablation of atrial fibrillation—patient satisfaction from a single-center UK experience. Journal of Interventional Cardiac Electrophysiology. 2013;37(3):291-303.

18. Tevis SE, Kennedy GD, Kent KC. Is there a relationship between patient satisfaction and favorable surgical outcomes? Advances in surgery. 2015;49(1):221.

19. Al-Abri R, Al-Balushi A. Patient satisfaction survey as a tool towards quality improvement. Oman medical journal. 2014;29(1):3.

20. Shafer L, Walker J, Waldman C, Yang C, Michaud V, Bernstein C, et al. Factors associated with anxiety about colonoscopy: the preparation, the procedure, and the anticipated findings. Digestive Diseases and Sciences. 2018;63:610-8.

21. Lindsay AC, Bishop J, Harron K, Davies S, Haxby E. Use of a safe procedure checklist in the cardiac catheterisation laboratory. BMJ Open Quality. 2018;7(3):e000074.

22. Hasballah SM, Shaor OAE, Mohamed MA, Mohamed AK. Assess nurses' knowledge and attitude for patient safety in cardiac catheterization unit. Assiut Scientific Nursing Journal. 2019;7(19):151-9.

23. AFKAR RM, SALMA GM, ELSISI AM, MARWA AI. Effect of Nursing Guidelines for Nurses on Occurrence of Selected Post Therapeutic Cardiac Catheterization Complications Among Children. The Medical Journal of Cairo University. 2018;86(June):2127-39.

24. Hassan C, East J, Radaelli F, Spada C, Benamouzig R, Bisschops R, et al. Bowel preparation for colonoscopy: European Society of Gastrointestinal Endoscopy (ESGE) guideline–update 2019. Endoscopy. 2019;51(08):775-94.

25. [Available from: https://www.equator-network.org/reporting-guidelines/consort/.

26. Aggarwal R, Mytton OT, Derbrew M, Hananel D, Heydenburg M, Issenberg B, et al. Training and simulation for patient safety. BMJ Quality & Safety. 2010;19(Suppl 2):i34-i43.

27. Bott KA. Effect of Enhanced Patient Education on Patient Satisfaction and 30-Day Readmission Rates after Cardiac Arrhythmia Ablation. 2016.

28. Lutjeboer J, Burgmans MC, Chung K, van Erkel AR. Impact on Patient Safety and Satisfaction of Implementation of an Outpatient Clinic in Interventional Radiology (IPSIPOLI-Study): a quasi-experimental prospective study. Cardiovascular and interventional radiology. 2015;38(3):543-51.

29. Cannon C, editor Telehealth, mobile applications, and wearable devices are expanding cancer care beyond walls. Seminars in oncology nursing; 2018: Elsevier.

30. Athilingam P, Osorio RE, Kaplan H, Oliver D, O'neachtain T, Rogal PJ. Embedding patient education in mobile platform for patients with heart failure: theory-based development and beta testing. CIN: Computers, Informatics, Nursing. 2016;34(2):92-8.

31. Kahn JG, Yang JS, Kahn JS. 'Mobile'health needs and opportunities in developing countries. Health affairs. 2010;29(2):252-8.

32. Thakkar J, Kurup R, Laba T-L, Santo K, Thiagalingam A, Rodgers A, et al. Mobile telephone text messaging for medication adherence in chronic disease: a meta-analysis. JAMA internal medicine. 2016;176(3):340-9.

33. Skinner C, Finkelstein J, editors. Review of mobile phone use in preventive medicine and disease management. Proceedings of the IASTED International Conference on Telehealth/Assistive Technologies; 2008: ACTA Press.

34. Liu C, Zhu Q, Holroyd KA, Seng EK. Status and trends of mobile-health applications for iOS devices: A developer's perspective. Journal of Systems and Software. 2011;84(11):2022-33.

Supporting information:

Fig 1. CONSORT flowchart for the study

Table 1. Participants' demographic characteristics

Table 2. Comparative analysis of patient satisfaction between the control and intervention groups

research ethics committees certificate. ENGLISH

Click here to access/download
Supporting Information
ethics committee. ENGLISH.pdf

research ethics committees certificate. PERSIAN

Click here to access/download
Supporting Information
ethics committee. PERSIAN.pdf

CONSORT. Fig 1.

Click here to access/download
Supporting Information
CONSORT Flow Diagram.doc

IRCT. Persian

Click here to access/download
Supporting Information
IRCT.pdf

Click here to access/download
Supporting Information
PROPOSAL. Negin Farid.doc

CONSORT checklist

Click here to access/download
Supporting Information
CONSORT_checklist.docx

Table 1. Participants' demographic characteristics.

Click here to access/download
Supporting Information
Table 1.docx

Table 2. Comparative analysis of patient satisfaction between the control and intervention groups.

Click here to access/download
Supporting Information
Table 2.docx

IRCT English

Click here to access/download
Supporting Information
IRCT English.pdf

~~evaluation~~ Evaluation of the effect of reinforced education on the satisfaction of patients undergoing colonoscopy: a randomized controlled trial

Negin Farid[1*], Shakila Sharifian[1], Raziyeh Ghafouri[2]

[1] Student Research Committee, School of Nursing & Midwifery, Shahid Beheshti University of Medical Sciences, Tehran, Iran

[2] Department of Medical and Surgical, School of Nursing & Midwifery, Shahid Beheshti University of Medical Sciences, Tehran, Iran

*Corresponding author:

~~Negin Farid~~

E-mail: neginfarid.79@gmail.com, n.farid@sbmu.ac.ir (NF)

Formatted: Line spacing: Double
Formatted: Highlight
Formatted: Highlight
Formatted: Highlight
Formatted: Highlight
Formatted: Highlight
Formatted: Highlight
Formatted: Highlight
Formatted: Highlight
Formatted: Highlight
Formatted: Highlight
Formatted: Highlight
Formatted: Highlight
Formatted: Font: Not Bold
Formatted: Font: Not Bold, Highlight
Formatted: Font: Not Bold
Formatted: Font: Not Bold
Formatted: Font: (Default) +Headings CS (Times New Roman)
Formatted: Font: (Default) +Headings CS (Times New Roman), 12 pt
Formatted: Font: (Default) +Headings CS (Times New Roman), Not Bold
Formatted: Font: (Default) +Headings CS (Times New Roman)
Formatted: Justified, Line spacing: Double
Formatted: Left, Line spacing: Double
Formatted: Font: (Default) +Headings CS (Times New Roman), Highlight
Formatted: Font: (Default) +Headings CS (Times New Roman)
Formatted: Default Paragraph Font, Font: (Default) +Headings CS (Times New Roman), 12 pt
Formatted: Font: (Default) +Headings CS (Times New Roman), 12 pt
Formatted: Default Paragraph Font, Font: (Default) +Headings CS (Times New Roman), 12 pt
Formatted: Font: (Default) +Headings CS (Times New Roman), 12 pt
Formatted: Font: (Default) +Headings CS (Times New Roman), 12 pt, Highlight
Formatted: Line spacing: Double

This study was registered using code IRCT ID IRCT20210131050189N5 in the Iranian Registry of Clinical Trials.~~Tel: 0989120061201~~

~~Address: Vali-Asr Avenue, the intersection of Vali-Asr Avenue and Hashemi Rafsanjani (Neiaiesh) Highway, opposite Rajaee Heart Hospital, Tehran.~~

Formatted: Font: (Default) +Headings CS (Times New Roman), Highlight

Formatted: Font: (Default) +Headings CS (Times New Roman), Not Bold

Formatted: Font: (Default) +Headings CS (Times New Roman)

Formatted: Left, Line spacing: Double

Formatted: Font: (Default) +Headings CS (Times New Roman)

~~full~~ Full title: Evaluation of the effect of reinforced education on the satisfaction of patients undergoing colonoscopy: a randomized controlled trial ~~evaluation of the effect of reinforced education on the satisfaction of patients undergoing colonoscopy: a randomized trial~~

Formatted: Line spacing: Double

Short title: ~~effect~~ Effect of reinforced education on the satisfaction of patients undergoing colonoscopy

Formatted: Highlight

Formatted: Highlight

Formatted: Highlight

Formatted: Highlight

Formatted: Highlight

Formatted: Highlight

Formatted: Highlight

Formatted: Font: 18 pt, Highlight

Formatted: Font: 16 pt, Highlight

Formatted: Font: 16 pt

Abstract

Introduction

Fourteen million colonoscopies are performed annually in the United States, where the colonoscopy results depend on bowel preparation before the procedure. However, suitable preparation is not performed in 20-25% of cases, resulting in increased loss of time, failure to diagnose, procedure repetition, and decreased patient satisfaction. Therefore, this study was performed to evaluate the effect of reinforced education (RE) on the satisfaction of patients undergoing colonoscopy.

Methods

Formatted: Font: 16 pt, Highlight

The current study applied an experimental research method. Participants in the study included 83 patients undergoing colonoscopy referred to Ayatollah Taleghani Medical Educational Center, divided into control and intervention groups. ~~Data from the control group were collected first to prevent the dissemination of information between the two groups.~~ In the control group, standard education was administered, whereas in the intervention group, the

patient was instructed using media and virtually, and preparation was followed up the day before the colonoscopy. The research tool was a demographic and satisfaction questionnaire developed by the researcher. Analyses were conducted using IBM SPSS software (v. 20).

Results

Eighty-three patients, including 47 men and 36 women with an average age of ~~19.49~~49.19 years, participated in the study. Mean (standard deviation) patient satisfaction was 11.78 (4.65) in the intervention group and 9.04 (2.95) in the control group; the independent t-test revealed a significant difference between the two groups ($P<0.001$).

Conclusions

The study suggests that reinforced education using media and virtual means is effective in the satisfaction of patients undergoing colonoscopy.

Keywords*:* colonoscopy; satisfaction; bowel preparation; reinforced education

Formatted: Font: 16 pt, Highlight

Formatted: Highlight

Formatted: Highlight

Formatted: Font: 16 pt, Highlight

Formatted: Font: 16 pt, Not Italic, Highlight

Introduction

Formatted: Font: 18 pt, Highlight

Formatted: Font: 18 pt

Fourteen million colonoscopies are performed annually in the United States (1), where colonoscopy results depend on bowel preparation before the procedure (1, 2). Inadequate preparation fails to diagnose 42% of adenomas and 27% of advanced adenomas. Proper colon preparation facilitates a more thorough examination of the mucosa, the presence of polyps and adenomas, and early cancer diagnosis (1). In 20-25% of cases, however, adequate preparation is lacking (3), resulting in an increased loss of time, failure to diagnose, an increase in cost (3, 4), and a higher risk of procedural adverse events, such as bleeding or perforation (4).

Although proper bowel preparation is the most important factor in colonoscopy (3), patients frequently find it uncomfortable (5). One in five individuals fails to take the prescribed dose of colonoscopy preparation drugs (1). Gender, constipation, drug use, previous colon surgery, diabetes, obesity, neurological disease, and lack of knowledge influence inadequate preparation (3). Examining the patient's needs and educating them on the significance of colonoscopy preparation is one of the primary obstacles (3). Consequently, effective education can increase a patient's willingness to comply with instructions (1).

Education is provided through various methods, such as pamphlets, animations, messages, and videos, which increase patient readiness by 11-36% (2). If multiple instructional strategies are employed in education, they will be more effective (6). Furthermore, the training will be more influential if it is based on problem-solving techniques, the immediate need of the patient, and an appreciation of its significance (7).

Due to the significance of patient compliance and bowel preparation in colonoscopy procedures, training can be effective if it is patient-centered (8), interactive (5), individually focused (3) based on the patient's needs, and one that emphasizes the importance of bowel preparation (9). Patient acceptance is improved by all educational methods, including oral and

written education (3), face-to-face education, and media-based education (10). However, new methods such as utilizing technology, media (1), web applications (11), 3D online programs (12), and mobile applications (13, 14) may increase patient acceptance to a greater extent, necessitating additional research on their efficacy (1). Media use can make reinforced education (RE) more effective from admission until the colonoscopy day (15).

Formatted: Highlight
Formatted: Highlight
Formatted: Highlight
Formatted: Highlight
Field Code Changed

The level of patient satisfaction is a crucial criterion for evaluating the quality of service (16). Patient satisfaction is an indicator of the quality of care, which is a vital consideration when selecting a treatment center; therefore, it is essential to conduct a study to determine how to improve the quality of services and patient satisfaction (17). More research must be conducted to boost care recipients' satisfaction (18, 19). Patient dissatisfaction is caused by insufficient preparation, incorrect diagnosis, and repeated colonoscopies, which can be remedied through proper training and follow-up. Based on the information above, it can be concluded that patient satisfaction and bowel preparation can be improved using RE. Therefore, this study evaluated the effect of RE on the satisfaction of colonoscopy patients.

Methods

Formatted: Font: 18 pt, Highlight
Formatted: Font: 18 pt

This semi-experimental study evaluated the impact of using media and virtual means to educate Ayatollah Taleghani Medical Educational Center-referred colonoscopy patients. This medical center performs approximately 2,500 colonoscopies annually, or nearly 200 per month. The study's statistical population consisted of colonoscopy patients referred to the Ayatollah Taleghani Educational Medical Center, where the study sample comprised patients who met the inclusion criteria.

The study was registered with the Iranian Clinical Trial Registry (IRCT20210131050189N5). The authors confirm that all ongoing and related trials for this intervention are registered.

The study was registered with the Iranian Clinical Trial Registry (IRCT20210131050189N5). The authors confirm that all ongoing and related trials for this intervention are registered.

It should be mentioned that due to the type of intervention of this study, we were only satisfied with obtaining the code of ethics at the beginning. But after taking action to submit the article, and with a more detailed study of the WHO guidelines, we realized the necessity of registering our protocol in the trials registration center. Therefore, retrospectively and by obtaining the necessary permits, the trial was successfully registered.

~~The sampling period lasted from May 22 to June 22 (According to Iranian SH: Khordad 1st to Tir 1st). The inclusion criteria were the absence of movement, hearing, and vision disorders, a medical history free of cognitive diseases and depression, the non-use of neuroleptics, the absence of a recent tragedy such as the death of a loved one or hospitalization, and access to online resources and educational videos. Patients who experienced a new tragedy during the research or refused to continue cooperating were excluded.~~

Sample size/power:

The number of samples in each group was determined by the following equation:

$$n \geq 2\frac{\left(z_{\alpha/2} + z_{\beta}\right)^2 \sigma^2}{(\mu_1 - \mu_2)}$$

Probability of type 1 error: $\alpha = 0.05 \rightarrow z_{\alpha/2} = 1.96$

Probability of type 2 error: $\beta = 0.10 \rightarrow z_{\beta} = 1.28$

Formatted: Highlight

Formatted: Line spacing: Double

Formatted: Font: 16 pt, Bold, Font color: Auto

Formatted: Indent: First line: 0", Space After: 8 pt, Line spacing: Double

Formatted: Line spacing: Double

Power of observed effect size: $(\mu_1 - \mu_2)/\sigma = 0.75$

Minimum required sample: $n = 2(1.96 + 1.28)^2 \left(\frac{1}{0.75}\right)^2 = 37$

Each group required a minimum of 37 participants. Considering a potential 40% decline in population, the sample size for each group consisted of 50 patients.

Data collection and sampling:

The sampling period lasted from May 22 to June 22. Data collection was completed through August 7. The inclusion criteria were the absence of movement, hearing, and vision disorders, a medical history free of cognitive diseases and depression, the non-use of neuroleptics, the absence of a recent tragedy such as the death of a loved one or hospitalization, and access to online resources and educational videos. Patients who experienced a new tragedy during the research or refused to continue cooperating were excluded.

Red and blue colored cards were used to divide the participants into the control and intervention groups (Figure 1). *RG* designed the allocation sequence, *NF* and *SS* enrolled participants, and *NF* and *RG* assigned interventions to each group (*capital letters indicate the authors' initials*). Also First, the data from the control group were collected, and the patients in each group were blinded to their group classification.

The two groups were homogeneous, where no statistically significant differences were observed in age, sex, education level, underlying disease, or reason for admission ($P<0.05$).

Please insert Figure 1, CONSORT flowchart for the study (CONSORT) here.

Data collection tool:

- **Formatted:** Font: 16 pt, Bold, Font color: Auto, Pattern: Clear (White), Highlight
- **Formatted:** Indent: First line: 0", Line spacing: Double
- **Formatted:** Space After: 8 pt, Line spacing: Double
- **Formatted:** Font:
- **Formatted:** Line spacing: Double
- **Formatted:** Highlight
- **Formatted:** Font: Italic, Highlight
- **Formatted:** Highlight
- **Formatted:** Font: Italic, Highlight
- **Formatted:** Highlight
- **Formatted:** Font: Italic, Highlight
- **Formatted:** Highlight
- **Formatted:** Font: Italic, Highlight
- **Formatted:** Highlight
- **Formatted:** Font: Italic, Highlight
- **Formatted:** Highlight
- **Formatted:** Font: Italic, Highlight
- **Formatted:** Space After: 8 pt, Line spacing: Double
- **Formatted:** Indent: First line: 0", Line spacing: Double
- **Formatted:** Font: Bold, Highlight
- **Formatted:** Highlight
- **Formatted:** Font: Bold, Highlight
- **Formatted:** Line spacing: Double
- **Formatted:** Font: Bold
- **Formatted:** Font: 16 pt, Bold, Font color: Auto, Pattern: Clear (White), Highlight

Bisacodyl tablets and Pidrolax powder were used for intestinal preparation. The standard training method was provided to the control group, who received a printed training sheet. Patients in the intervention group were contacted the day before their colonoscopy. Their questions were answered, and training files, including videos, applications, and an Instagram page, were sent to them via WhatsApp.

Formatted: Indent: First line: 0.59", Line spacing: Double

The data collection tool included a demographic information questionnaire with 20 questions, including the patient's age, sex, level of education, marital status, occupation, type of health insurance, the reason for hospitalization, length of hospitalization, illness history, underlying disease, and medical family history, previous colonoscopy experience, place of residence, diet (daily fruit consumption, weekly fast food consumption), ability to perform daily activities, and amount of exercise per day..

Formatted: Highlight

The colonoscopy satisfaction instrument was a researcher-made instrument with 12 questions in 3 dimensions (pre-colonoscopy education, colonoscopy experience, and colonoscopy result). Before preparing the tool, its components were developed based on a review of the relevant literature (20-23). . Then, duplicate items were eliminated, categorized, and summarized by the expert panel consisting of ten gastroenterologists and endoscopy nursing experts. Next, face validity (qualitative), content validity (quantitative and qualitative), and construct validity were examined. Ten nurses were asked to provide their corrections regarding the use of correct grammar, vocabulary, placement of expressions, and provision of sufficient points to verify the accuracy of the content. The content validity index (CVI) and content validity ratio (CVR) were then determined and validated. The relationship between the items was then determined and summarized using exploratory factor analysis into three factors (pre-colonoscopy education, colonoscopy experience, and colonoscopy result). Cronbach's alpha was used to test reliability. Cronbach's alpha of the instrument was 0.83.

Formatted: Line spacing: Double

Formatted: Highlight

Formatted: Highlight

Formatted: Highlight

The colonoscopy patients were contacted one week after the procedure, and the aforementioned patient satisfaction questionnaire was administered.

Intervention:

The standard training program was provided to the control group, which received a printed training sheet. In the intervention group, patients were contacted the day before their colonoscopy, and training materials, including video clips, an application, and an Instagram page, were sent to them via the WhatsApp application, and their questions were answered.

Bisacodyl tablets and Pidrolax powder were utilized for Bowel preparation. Below are the morning and afternoon versions of the routine protocol for bowel preparation (as recommended by ESGE (24)).

Colonoscopy appointment between 8:00 to 13:00

One day before a colonoscopy, only liquids, including water, sugar water, compote water, tea, strained fruit juice (clear and pulp-free), meat broth, and chicken broth, may be consumed beginning at 9 am. Avoid soups, dairy products, juices containing fruit particles, compotes, and solid foods such as (bread, rice, potatoes, meat, and ash).

Maintain a fluid-only diet throughout the day. Use one tablet of Bisacodyl at noon, 4 pm, and 8 pm.

Use six colonoscopy preparation powders (Pidrolox) between noon and midnight. Each powder is mixed with one liter of water (four large glasses), and one glass is consumed every half-hour.

At least three to four hours of walking and physical activity are required daily.

Start fasting in the morning on the day of the test.

Colonoscopy appointment between 13:00 to 19:00

One day before the colonoscopy, only liquids, including water, sugar water, compote water, tea, strained fruit juice (clear and pulp-free), meat broth, and chicken broth, may be consumed

Formatted: Font: +Headings CS (Times New Roman), 16 pt, Bold, Pattern: Clear (White), Highlight

Formatted: Indent: First line: 0", Line spacing: Double

beginning at 9:00 am. Avoid soups, dairy products, fruit juices containing fruit particles, compotes, and solid foods such as (bread, rice, potatoes, meat, and ash).

Maintain a fluid-only diet throughout the day. Use one tablet of Bisacodyl at noon, 4:00, and 8:00 pm.

Use five powders for colonoscopy preparation between 4:00 pm and 12:00 am. Pour each powder into one liter of water (four large glasses), and consume one glass every 20 minutes.

At least three to four hours of walking and physical activity are required daily.

At 8:00 am on the colonoscopy day, a preparation powder should be mixed with one liter of water (four large glasses) and consumed. Fasting begins at 10 am.

Formatted: Indent: First line: 0", Line spacing: Double

~~(24)~~Data analysis:

Formatted: Font: +Headings CS (Times New Roman), 16 pt, Bold, Pattern: Clear (White), Highlight

IBM SPSS software was employed for analyses (v. 20, IBM Corp., Armonk, NY). Kolmogorov-Smirnov tests were used to assess the normality of the distribution of variables. A p-value < 0.05 was deemed statistically significant. Either the t-test or the chi-square test was utilized to analyze demographic data. If the data distribution was normal, the paired t-test and student t-test were used to compare variables within and between groups.

Formatted: Line spacing: Double

Notably, we used the 2010 version of the CONSORT reporting guidelines for this manuscript (25).

Results

Formatted: Font: 18 pt, Highlight

Formatted: Font: 18 pt

In this study, 83 patients with an average age of 49.19 years participated, including 47 men and 36 women. Table 1 shows the demographic characteristics of the intervention group (43 individuals) and the control group (40 individuals).

Table 1. Participants' demographic characteristics. ~~Please insert Table 1 here.~~

Formatted: Highlight

Formatted: Left, Line spacing: Double

		Group			
		Control		Intervention	
		Count	N %	Count	N %
Gender	Female	18	21.7%	18	21.7%
	Male	25	30.1%	22	26.5%
Marital Status	Single	7	8.6%	6	7.4%
	Marriage	36	44.4%	32	39.5%
Education	School	17	21.5%	15	19.0%
	High School	19	24.1%	16	20.3%
	Bachelor	2	2.5%	4	5.1%
	Upper	2	2.5%	4	5.1%
Number Colonoscopy	First	29	34.9%	19	22.9%
	Second	10	12.0%	10	12.0%
	More	4	4.8%	11	13.3%
Hospitalization	Outpatient	33	39.8%	35	42.2%
	Inpatient	10	12.0%	5	6.0%
Family History	No	33	40.2%	29	35.4%

Formatted: Highlight

Formatted: Highlight

Formatted: Highlight

Formatted: Highlight

	Yes	10	12.2%	10	12.2%

Initially, we examined the homogeneity of the two groups and observed no significant difference (P>0.05). Mean (standard deviation) patient satisfaction was 11.78 (4.65) in the intervention group and 9.04 (2.95) in the control group. The independent t-test revealed a statistically significant difference between the two groups (P<0.001). In the colonoscopy experience factor, the t-test revealed that the difference was not statistically significant (P>0.05). In the colonoscopy results (P=0.05); and pre-colonoscopy education, the t-test indicated that the difference was statistically significant (P<0.001). ~~(P<0.01). In the colonoscopy experience factor, the t-test indicated that the difference was insignificant; however, the t-test revealed a significant difference (P<0.05) in the patient satisfaction, colonoscopy results, and pre-colonoscopy education factors.~~ The results of patient satisfaction factors between the control and intervention groups are shown in Table 2.

Table 2. Comparative analysis of patient satisfaction between the control and intervention groups.~~Please insert Table 2 here.~~

	Control		Intervention			
	Mean	Standard Deviation	Mean	Standard Deviation	T	P value
pre-colonoscopy education	1.66	1.68	3.55	2.51	-3.99	0.00
colonoscopy experience	4.57	2.17	4.95	2.13	-0.68	0.49

Formatted: Line spacing: Double

Formatted: Highlight

Formatted: Font: Bold, Highlight

Formatted: Font: (Default) Calibri, 11 pt, Bold

Formatted: Justified, Line spacing: Double

Formatted: Line spacing: Double

Formatted: Line spacing: Double

Formatted: Line spacing: Double

Formatted: Line spacing: Double

colonoscopy result	1.30	0.66	1.63	0.77	-1.99	0.05

Discussion

The present study aimed to evaluate the effect of RE on the level of satisfaction of patients undergoing colonoscopy. The results indicated that RE using media and virtual means improved the level of satisfaction of these patients (P<0.01). Guo et al. observed similar effects of RE on bowel preparation (9). ~~(9)~~ In this regard, Aggarwal et al. have studied the effect of training on safety, stated that effective training methods should be utilized concerning patient safety, and emphasized the importance of continuing training (26). Bott et al. compared the impact of implementing a specialized training program after treating cardiac arrhythmia to standard discharge instructions in their study. According to the study's findings, the training provided by the nurse to the patient 24 to 72 hours after discharge increased patient satisfaction and reduced readmission rates (27). The results of Bott et al. are consistent with the current study.

In this regard, Lutjeboer et al. demonstrated that promoting self-care can affect and increase patient satisfaction. Their study was conducted in the radiology department (28). In the present study, patient-centered training increased patient satisfaction. Similarly, Ezzat et al. found that reducing complications during cardiac catheterization can increase patient satisfaction (17). Since the reduction of complications is a component of self-care training, their results are also consistent with those of the present study and indicate a rise in satisfaction following the promotion of self-care with training and follow-up of care and preparations.

Patient education is crucial for promoting self-management and decreasing hospitalization and treatment costs (27). Therefore, three to four dollars are saved in costs for

Formatted: Line spacing: Double

Formatted: Line spacing: Double

Formatted: Font: 18 pt, Highlight

Formatted: Font: 18 pt

Formatted: Highlight

every dollar spent on patient education. According to presented statistics, approximately 69 to 100 million dollars are spent annually in the United States on medical issues resulting from a lack of education (28).

The majority of individuals possess cell phones today (29). Athilingam stated in 2016 that there are 7 billion cell phones worldwide and that nearly 95% of the global population uses cell phones (30). The owner's cell phone is with them throughout the day (30), and its use is beneficial for teaching self-care and disease prevention programs (31). Utilization and accessibility have increased satisfaction (32). The cell phone is one of the most widely used technological innovations that improve communication between the patient and the medical staff in the health system, and with suitable programs, higher attractiveness, and the possibility of remote patient follow-up, it has contributed to patient satisfaction (33, 34). The current study used mobile and media programs to improve patient education and follow-up. Mobile education in the reinforced educational program was more acceptable and increased patient satisfaction.

Even though our study group was limited to patients undergoing colonoscopy, and this study was conducted with random samples from an Iranian population, and should allude to future studies with much larger sample sizes collected at other geographical locations to confirm the efficacy of the RE procedure, it appears that the result can be generalized to other procedures where the background knowledge of the patient and their family is incomplete and limited, because it reduplicates the importance of the patient's and family's participation in the decision-making process.

~~it appears that the results can be generalized to other procedures where the patient and their family have incomplete and limited background knowledge because it reduces the fear of the unknown in a novel and understandable manner.~~

Research Limitations

Formatted: Highlight

Formatted: Highlight

Formatted: Highlight

Formatted: Highlight

Formatted: Highlight

Formatted: Highlight

Formatted: Highlight

Formatted: Font: 14 pt, Highlight

Formatted: Font: 12 pt

The outbreak of COVID-19, which hindered data collection, was one of the study's limitations; however, the use of virtual means and telephone follow-up of patients did not affect the findings. One of the strengths was the use of several teaching methods.

Conclusions

Formatted: Font: 18 pt, Highlight

Formatted: Font: 18 pt

Based on the results, it can be concluded that through media and cyberspace utilization, RE is effective in the satisfaction of patients undergoing colonoscopy. It is important to note that the current findings are based solely on random samples derived from the Iranian population and that future studies with much larger sample sizes collected from other geographic regions are required to confirm the efficacy of the RE method.

Formatted: Highlight

~~Based on the findings, it is possible to conclude that reinforced education through the use of media and performed virtually is effective in increasing the satisfaction of patients undergoing colonoscopy.~~

Formatted: Font: 12 pt

List of abbreviations

Formatted: Font: 14 pt, Highlight

Formatted: Font: 14 pt

Formatted: Highlight

RE: Reinforced Education

CVR: Content Validity Ratio

CVI: Content Validity Index

3D: ~~3~~ Three Dimensions

Formatted: Line spacing: Double

Acknowledgments

Formatted: Font: 14 pt, Highlight

Formatted: Highlight

Formatted: Font: 14 pt, Bold

We would like to thank all participants and those who assisted us in conducting the research, particularly the entire staff of the colonoscopy department at Ayatollah Taleghani Educational

Medical Center and the Gastroenterology and Liver Diseases Research Center at the Research Institute for Gastroenterology and Liver Diseases of Shahid Beheshti University of Sciences.

~~We would like to thank all participants and those who assisted us in performing the research, especially the Ayatollah Taleghani Educational Medical Center's colonoscopy department staff.~~

Formatted: Line spacing: Double

Authors' contributions

Formatted: Font: 14 pt, Highlight

Formatted: Font: 14 pt

NF and RG conceived and designed the study.

NF, SS, and RG developed methodology.

NF, SS, and RG gathered, analyzed, and interpreted the data.

NF and RG wrote and revised the manuscript.

RG supervised and coordinated all aspects of the work.

All authors have read and approved the manuscript.

Funding

Formatted: Font: 14 pt, Highlight

Formatted: Font: 14 pt

We did not receive external funding for this research.

Consent for publication

Formatted: Font: 14 pt, Highlight

Formatted: Font: 14 pt

Not applicable.

Availability of Data and Materials:

Formatted: Font: 14 pt, Highlight

Formatted: Font: 14 pt

All data generated or analyzed during this study are included in this published article and its supplementary file.

Ethical Consideration

Formatted: Font: 14 pt, Highlight

Formatted: Font: 14 pt

Formatted: Font: 14 pt, Bold

The Ethics Committee of the Student Research Committee at Shahid Beheshti University of Medical Sciences approved the study protocol (IR.SBMU.RETECH.REC. 1400.1082) entitled "Evaluation of the effect of reinforced education with media on patient education on colonoscopy satisfaction." To adhere to ethical considerations, the research objectives and methods were explained to the participants, they were assured of the anonymity and confidentiality of their information, and each participant provided written informed consent. They participated voluntarily and had the option to withdraw at any time. This research was registered with IRCT ID IRCT20210131050189N5 in the Iranian Registry of Clinical Trials.

Competing interests

The authors declare that they have no competing interests and confirm that all ongoing and related trials for this intervention are registered.

;

References

1. Kurlander JE, Sondhi AR, Waljee AK, Menees SB, Connell CM, Schoenfeld PS, et al. How efficacious are patient education interventions to improve bowel preparation for colonoscopy? A systematic review. PloS one. 2016;11(10):e0164442.

2. Jeon SC, Kim JH, Kim SJ, Kwon HJ, Choi YJ, Jung K, et al. Effect of sending educational video clips via smartphone mobile messenger on bowel preparation before colonoscopy. Clinical endoscopy. 2019;52(1):53.

3. Elvas L, Brito D, Areia M, Carvalho R, Alves S, Saraiva S, et al. Impact of personalised patient education on bowel preparation for colonoscopy: prospective randomised controlled trial. GE-Portuguese Journal of Gastroenterology. 2017;24(1):22-30.

Formatted: Font: 14 pt, Highlight

Formatted: Font: 14 pt

Formatted: Highlight

Formatted: Line spacing: Double

Formatted: Font: 18 pt, Highlight

Formatted: Font: 18 pt

Formatted: Font: Do not check spelling or grammar

Formatted: Justified, Line spacing: Double

4. Sadeghi A, Rajabnia M, Bagheri M, Jamshidizadeh S, Saberi S, Shahnazi P, et al. Predictive factors of inadequate bowel preparation for elective colonoscopy. Gastroenterology and Hepatology From Bed to Bench. 2022;15(1):66.

5. Schooley B, San Nicolas-Rocca T, Burkhard R. Cloud-based multi-media systems for patient education and adherence: a pilot study to explore patient compliance with colonoscopy procedure preparation. Health Systems. 2021;10(2):89-103.

6. Marble N, Loescher LJ, Lim KH, Hiscox H. Use of technology for educating melanoma patients. Journal of Cancer Education. 2010;25(3):445-50.

7. Cook DJ, Moradkhani A, Douglas KSV, Prinsen SK, Fischer EN, Schroeder DR. Patient education self-management during surgical recovery: combining mobile (iPad) and a content management system. Telemedicine and e-Health. 2014;20(4):312-7.

8. Bernstein MT, Kong J, Sriranjan V, Reisdorf S, Restall G, Walker JR, et al. Evaluating information quality of revised patient education information on colonoscopy: It is new but is it improved? Interactive journal of medical research. 2019;8(1):e11938.

9. Guo X, Li X, Wang Z, Zhai J, Liu Q, Ding K, et al. Reinforced education improves the quality of bowel preparation for colonoscopy: An updated meta-analysis of randomized controlled trials. PloS one. 2020;15(4):e0231888.

10. Bernstein MT, Garber J, Faucher P, Reynolds KA, Restall G, Walker JR, et al. New Patient Education Video on Colonoscopy Preparation: Development and Evaluation Study. JMIR human factors. 2020;7(4):e15353.

11. Trasolini R, Nap-Hill E, Suzuki M, Galorport C, Yonge J, Amar J, et al. Internet-based patient education prior to colonoscopy: Prospective, observational study of a single center's implementation, with objective assessment of bowel preparation quality and patient satisfaction. Journal of the Canadian Association of Gastroenterology. 2020;3(6):274-8.

12. Zhao Y, Xie F, Bai X, Yang A, Wu D. Educational virtual reality videos in improving bowel preparation quality and satisfaction of outpatients undergoing colonoscopy: protocol of a randomised controlled trial. BMJ open. 2019;9(8):e029483.

13. Walter B, Schmid R, von Delius S. A smartphone app for improvement of colonoscopy preparation (ColoprAPP): development and feasibility study. JMIR mHealth and uHealth. 2017;5(9):e7703.

14. Cho J, Lee S, Shin JA, Kim JH, Lee HS. The impact of patient education with a smartphone application on the quality of bowel preparation for screening colonoscopy. Clinical Endoscopy. 2017;50(5):479-85.

15. Walter B, Klare P, Strehle K, Aschenbeck J, Ludwig L, Dikopoulos N, et al. Improving the quality and acceptance of colonoscopy preparation by reinforced patient education with short message service: results from a randomized, multicenter study (PERICLES-II). Gastrointestinal endoscopy. 2019;89(3):506-13. e4.

16. Ghazi K, Foroughan M, Hosseini M, Hosseinzadeh S, Askari M. The client satisfaction of delivered services in private nursing homes for elderly: a survey in the provinces of Golestan, Mazandaran, Semnan and Northern Khorasan in 2012 or 1391. Journal of Sabzevar University of Medical Sciences. 2013;20(3):320-30.

17. Ezzat VA, Chew A, McCready JW, Lambiase PD, Chow AW, Lowe MD, et al. Catheter ablation of atrial fibrillation—patient satisfaction from a single-center UK experience. Journal of Interventional Cardiac Electrophysiology. 2013;37(3):291-303.

18. Tevis SE, Kennedy GD, Kent KC. Is there a relationship between patient satisfaction and favorable surgical outcomes? Advances in surgery. 2015;49(1):221.

19. Al-Abri R, Al-Balushi A. Patient satisfaction survey as a tool towards quality improvement. Oman medical journal. 2014;29(1):3.

20. Shafer L, Walker J, Waldman C, Yang C, Michaud V, Bernstein C, et al. Factors associated with anxiety about colonoscopy: the preparation, the procedure, and the anticipated findings. Digestive Diseases and Sciences. 2018;63:610-8.

21. Lindsay AC, Bishop J, Harron K, Davies S, Haxby E. Use of a safe procedure checklist in the cardiac catheterisation laboratory. BMJ Open Quality. 2018;7(3):e000074.

22. Hasballah SM, Shaor OAE, Mohamed MA, Mohamed AK. Assess nurses' knowledge and attitude for patient safety in cardiac catheterization unit. Assiut Scientific Nursing Journal. 2019;7(19):151-9.

23. AFKAR RM, SALMA GM, ELSISI AM, MARWA AI. Effect of Nursing Guidelines for Nurses on Occurrence of Selected Post Therapeutic Cardiac Catheterization Complications Among Children. The Medical Journal of Cairo University. 2018;86(June):2127-39.

24. Hassan C, East J, Radaelli F, Spada C, Benamouzig R, Bisschops R, et al. Bowel preparation for colonoscopy: European Society of Gastrointestinal Endoscopy (ESGE) guideline–update 2019. Endoscopy. 2019;51(08):775-94.

25. [Available from: https://www.equator-network.org/reporting-guidelines/consort/.

26. Aggarwal R, Mytton OT, Derbrew M, Hananel D, Heydenburg M, Issenberg B, et al. Training and simulation for patient safety. BMJ Quality & Safety. 2010;19(Suppl 2):i34-i43.

27. Bott KA. Effect of Enhanced Patient Education on Patient Satisfaction and 30-Day Readmission Rates after Cardiac Arrhythmia Ablation. 2016.

28. Lutjeboer J, Burgmans MC, Chung K, van Erkel AR. Impact on Patient Safety and Satisfaction of Implementation of an Outpatient Clinic in Interventional Radiology (IPSIPOLI-Study): a quasi-experimental prospective study. Cardiovascular and interventional radiology. 2015;38(3):543-51.

29. Cannon C, editor Telehealth, mobile applications, and wearable devices are expanding cancer care beyond walls. Seminars in oncology nursing; 2018: Elsevier.

30. Athilingam P, Osorio RE, Kaplan H, Oliver D, O'neachtain T, Rogal PJ. Embedding patient education in mobile platform for patients with heart failure: theory-based development and beta testing. CIN: Computers, Informatics, Nursing. 2016;34(2):92-8.

31. Kahn JG, Yang JS, Kahn JS. 'Mobile'health needs and opportunities in developing countries. Health affairs. 2010;29(2):252-8.

32. Thakkar J, Kurup R, Laba T-L, Santo K, Thiagalingam A, Rodgers A, et al. Mobile telephone text messaging for medication adherence in chronic disease: a meta-analysis. JAMA internal medicine. 2016;176(3):340-9.

33. Skinner C, Finkelstein J, editors. Review of mobile phone use in preventive medicine and disease management. Proceedings of the IASTED International Conference on Telehealth/Assistive Technologies; 2008: ACTA Press.

34. Liu C, Zhu Q, Holroyd KA, Seng EK. Status and trends of mobile-health applications for iOS devices: A developer's perspective. Journal of Systems and Software. 2011;84(11):2022-33.

Supporting information:

Fig 1. CONSORT flowchart for the study

Table 1. Participants' demographic characteristics

Table 2. Comparative analysis of patient satisfaction between the control and intervention groups

Formatted: Line spacing: Double

Formatted: Font: 18 pt, Bold, Font color: Custom Color(RGB(34,34,34)), Highlight

Formatted: Font: 18 pt, Bold, Font color: Custom Color(RGB(34,34,34)), Highlight

Formatted: Font: 18 pt

Formatted: Font: Bold

Formatted: Left, Line spacing: Double

Formatted: Font: 14 pt, Bold

Response to Reviewers

Dear Dr. Brillantino

Thank you for giving us the opportunity to submit a revised draft of our manuscript titled [Evaluation of the effect of reinforced education on the satisfaction of the patients undergoing colonoscopy: a randomized controlled trial] to *PLOS ONE*. We appreciate the time and effort that you and the reviewers have dedicated to providing your valuable feedback in our manuscript. We are grateful to the reviewers for their insightful comments on our paper. We have been able to incorporate changes to reflect most of the suggestions provided by the reviewers.We have highlighted the changes within the manuscript.

Here is a point-by-point response to the reviewers` comments and concerns:

Reviewer Comments	Response to The Reviewer	Page in Manuscript
**Journal Requirements:**		
**1.** Ensure that your manuscript meets PLOS ONE's style requirements.	We checked the style approved by PLOS ONE following the two links sent in the email and reflected in our manuscript.<br>The changed parts are highlighted in the "track changes" file.	All of manuscript
**2-1.** your reasons for your delay in registering this study (after enrolment of participants started).	In fact, we did not know that obtaining the trial registration code is a necessity, so we settled for obtaining the code of ethics at first. After reading the guidelines and the recommendation of PLOS ONE, we registered our trial, which was successfully conducted and approved. (Documents previously attached for the journal)<br>For this reason, the registration of the trial was after obtaining the code of ethics and sampling procedures.	--
**2-2.** confirmation that all related trials are registered by stating.	We added it.	15

**3.** State the name of the registry and the registration number in the submission data and on the title page of your manuscript.	We added it.	1,5,15
**3-a.** provide the complete date range for participant recruitment and follow-up in the methods section of your manuscript.	We added it.	6
**3-b.** confirm that all related trials are registered by stating: "The authors confirm that all ongoing and related trials for this drug/intervention are registered".	We added it.	5,15
**4.** We note that the original protocol that you have uploaded as a Supporting Information file	The institutional logo of the original protocol was removed and uploaded again.	--

contains an institutional logo. As this logo is likely copyrighted, we ask that you please remove it from this file and upload an updated version upon resubmission.		
**5.** Amend either the abstract on the online submission.	Sure.<br>Done.	--
**6.** Include captions for your Supporting Information files at the end of your manuscript, and update any in-text citations to match accordingly.	We added it.	20
**Reviewer 1**:		
The scientific writing of the article is not enough.	With the help of *Tarjomic* Institute, we wrote the article natively and uploaded its certificate to the journal as supporting information.	--

File (by reviewer 1)	We are very grateful for your detailed and insightful comments, which greatly helped to improve the quality of our paper.<br>We have reviewed your comments one by one:<br>• If there was a typographical or spelling error, we corrected it. (Actually, we have not included this category of corrections below, but we have highlighted the corrections in the revised manuscript.)<br>• If you have asked a question, we have answered it in the text of the manuscript to make it clearer for the reviewers and readers.<br>• If the information provided by us was incomplete somewhere, we corrected and completed that part and matched it with your order or the article referred by you.<br>• Incomplete references were corrected and matched.<br>• Where our writing was ambiguous, we gave a clearer explanation.	

	All these items can be seen highlighted in the text of the article (track changes file).	
Title: randomized controlled trial	Corrected.	1,2
Abstract: Results: $p < 0.001$	Corrected.	3
The esge guideline recommendation should be appended with the level of evidence.	Our bowel preparation was in accordance with the ESGE guidelines, which were included in the text of the paper and added to the references.	8
What is the annual number of colonoscopies performed at Ayatollah Taleghani Medical Educational Center?<br><br>What is the average number of patients per month?	We added answers of these questions in the Methods Section.<br><br>"This medical center performs approximately 2,500 colonoscopies annually, or nearly 200 per month."	5

WHEN WAS THE DATA COLLECTED?	We answered this question completely in the Methods Section, data collection and sampling part.<br>"The sampling period lasted from May 22 to June 22. Data collection was completed through August 7.	6
How is the bowel preparation protocol routine?<br>What is the dose frequency and amount? How many days does it take? Is it just medical preparation?<br>No dietary restrictions?<br>When is oral intake stopped?	You are right and we did not explain about this. But now in the methods section, intervention part, we have fully described the routine protocol and its details.	8-9
When was the questionnaire administered and where was it administered?	We answered this question in the Methods Section, data collection part.<br>"Questionnaire questions were asked by the sampler to the sample people and entered into Google form at the same moment in the hospital."	7

Write down all the questions. 20 variables are not included here.	age, sex, level of education, marital status, occupation, type of health insurance, the reason for hospitalization, length of hospitalization, illness history, underlying disease, medical family history, previous colonoscopy experience, place of residence, diet (daily fruit consumption, weekly fast food consumption), ability to perform daily activities, and amount of exercise per day.consumption, weekly fast food consumption), ability to perform daily activities, amount of exercise per day.	7
Methods: Citations are added.	We added.	7
Methods: Endoscopy nurses or nurses?	endoscopy nursing experts	7
Table 2: Add the statistical significance criterion. If p < 0.05, this value (0.049) cannot be significant.	Yes, you`re right and 0.05 cannot be significant.	11

The studies used in the discussion cover different samples. Moreover, they did not receive training on bowel preparation. Does not match your goals (eg: length of stay etc.)<br><br>The discussion should be rewritten in relation to the findings. The sample should include colonoscopy patients, education bowel preparation instructions. Furthermore, the results should measure patient satisfaction.	Thanks for your consideration. Actually, these studies are presented in the discussion section due to their similarity in the topic of patient education and its effectiveness	11-12
I DID NOT UNDERSTAND THIS LIMITATION. BOTH PREPARATION INCLUDES	We explained this part more clearly at the end of the discussion section.	13

ONLY PATIENTS WITH A COLONOSCOPY ALREADY. WHAT DO YOU MEAN?		
WHEN WAS THE DATA COLLECTED? DID YOU COLLECT DATA DURING THE PANDEMIC?	Yes, and it is mentioned in the text.	13
**Reviewer 2:**		
**1. Methods:**		
An orderly manner is suggested. The authors are advised to create separate subsections for each of the possible topics (whichever necessary), and that way produce a very clear writeup.	We separated the method section into smaller components and completed the requirements according to the CONSORT 2010.	5-9

**a.** the randomization and allocation concealment should be made very clear (they are NOT the same thing); the trial staff recruiting patients should NOT have the randomization list. Randomization should be prepared by the trial statistician, and he/she would not participate in the recruiting	You are right, a researcher who don`t educated or collected data, recruiting patients.	--
**b**. The manuscript does not provide details on the randomization procedure; any reasoning, why a block randomization was not used, which is often recommended to ensure a balance in sample size across groups	That was about education and blinded for researcher who collected data.	--

**c.** The authors write in the Abstract: "In order to prevent the dissemination of information between the two groups, first the data of the control group was collected". This is not clear! You may easily attain information blockade between the 2 groups via blinding, and a proper randomization procedure. More details needed	We collected data in deferent days based on allocation.	--
**d.** Sample size/power: A sample size/power statement is made available, but its recommended to place it as a separate subsection within the Methods section	We corrected in.	6

**e-1.** Alternative analysis methods under data non-Gaussianity is not mentioned, such as using well-known nonparametric tests	We added it. Initially, we examined the homogeneity of the two groups and observed no significant difference (P>0.05).	11
**e-2.** Several additional covariates (via the questions) were also obtained. Why a formal regression analysis was not conducted?<br><br>**e-3.** It's not clear if data was collected longitudinally; please provide details. If that is the case, mixed modeling should be used.	The covariate and mixed model analysis was done considering the intervention and matching of the two groups. Added assimilation in sampling and results.	--
**e-4.** Overall write up has various inconsistencies; please fix incomplete sentences. For example.	We corrected them.	--

**2. Results & Conclusions:**		
**a.** The authors should check that any statement of significance should be followed by a p-value in the entire Results section.	We added it.	9-11
**b.** Conclusions should state that the current findings are ONLY based on the random samples derived from an Iranian population, and should allude to future studies with much larger sample sizes and collected at other geographical areas to confirm the effectiveness of the RE procedure.	We corrected it.	13-14
