# Supplementary material for: Evaluation of the effect of reinforced education on the satisfaction of patients undergoing colonoscopy: A randomized controlled trial
Source: PLoS One. 2024 Jan 5;19(1):e0296126. doi: 10.1371/journal.pone.0296126 (PMC10769074; doi:10.1371/journal.pone.0296126)
Supplement: Attachment — Submitted filename: REVIEWER1_Evaluation of the Effect of Reinforced Education on the Satisfaction of the Patients Undergoing Colonoscopy a Randomized Trial.docx [file pone.0296126.s003.docx]

**Evaluation of the Effect of Reinforced Education on the Satisfaction of the Patients Undergoing Colonoscopy: a Randomized Trial**

**Negin Farid^1^, Shakila Sharifian^1^, Raziyeh Ghafouri^2^**

^1^ Student Research Committee, School of Nursing & Midwifery, Shahid Beheshti University of Medical Sciences, Tehran, Iran

^2^ Department of Medical and Surgical, School of Nursing & Midwifery, Shahid Beheshti University of Medical Sciences, Tehran, Iran

**^*^Coresponding author:**

**Negin Farid**

Email: [neginfarid.79@gmail.com](mailto:neginfarid.79@gmail.com), [n.farid@sbmu.ac.ir](mailto:n.farid@sbmu.ac.ir)

Tel: 09120061201

Address: Vali-Asr Avenue, Cross of Vali-Asr Avenue and Hashemi Rafsanjani (Neiaiesh) Highway, Opposite to Rajaee Heart Hospital, Tehran.

**Full title: Evaluation of the Effect of Reinforced Education on the Satisfaction of the Patients Undergoing Colonoscopy: a Randomized Trial**

**Short title: Effect of Reinforced Education on the Satisfaction of the Patients Undergoing Colonoscopy**

**Abstract**

**Introduction:**

Annually, fourteen million colonoscopies are performed in The United States, and the colonoscopy results depend on bowel preparation before the procedure. However, in 20-25% of cases, good preparation is not done, which leads to more wasted time, failure to diagnose, repeat it, and decrease patient satisfaction. Therefore, this study was conducted with the aim of evaluating the effect of Reinforced Education on the satisfaction of the patients undergoing colonoscopy.

**Methods:**

This research was carried out in an experimental way. The participants in the study were 83 patients undergoing colonoscopy referred to Ayatollah Taleghani Medical Educational Center, who were divided into control and intervention groups. In order to prevent the dissemination of information between the two groups, first the data of the control group was collected. In the control group, education was given as usual, and in the intervention group, the patient was taught using media and cyberspace, and preparation was followed up on the day before the colonoscopy. The research tool was a demographic and satisfaction questionnaire made by the researcher. Analyses were conducted using IBM SPSS Statistics, Version 20.

**Results:**

83 patients including 47 men and 36 women with an average age of 19.49 years participated in the study. The mean (Standard Deviation) of patient satisfaction in the intervention group was 11.78 (4.65) and in the control group was 9.04 (2.95), the result of the independent t-test indicated a significant difference in the two groups (P<0 /01).

**Conclusions:**

 Based on the findings, it can be concluded that reinforced education using media and cyberspace is effective on the satisfaction of patients undergoing colonoscopy.

**Keywords:** colonoscopy; satisfaction; bowel preparation; reinforced education.

**Introduction**

Annually, fourteen million colonoscopies are performed in The United States (1), and the colonoscopy results depend on bowel preparation before the procedure (1, 2). 42% of adenomas and 27% of advanced adenomas are not diagnosed with inappropriate preparation. Proper preparation of the colon, helps to better observe the colon in terms of examining the mucosa, presence of polyps and adenomas, and early cancer diagnosis (1). However, in 20-25% of cases, good preparation is not done (3), which leads to more wasted time, failure to diagnose, increase in cost (3, 4) and increase the risk of procedural adverse events, such as bleeding or perforation (4).

Even though proper bowel preparation is the most important factor in colonoscopy (3), it is often uncomfortable for patients (5). One out of five people do not take the dose of colon preparation drugs before colonoscopy (1). Factors affecting incomplete preparation are; Gender, constipation, drug use, previous colon surgery, diabetes, obesity, neurological disease and lack of knowledge (3). One of the basic challenges is examining the patient's needs and teaching him/her the importance of colonoscopy preparation (3). Effective education can increase the patient's readiness to accept orders (1).

 Education is provided by various methods such as pamphlets, animations, messages and videos, which increase patient readiness by 11-36% (2). If several methods of teaching are used in education, it will be more effective (6). Also, if the training is based on the problem solving technique, the immediate need of the patient, understanding of its importance, it will be more impressive (7).

In colonoscopy procedures, due to the importance of patient compliance and bowel preparation, training can be effective if it is patient-centered (8), interactive (5) and individual based (3) on the patient's needs and emphasizing the importance of bowel preparation (9). All educational methods, including oral and written education (3), face-to-face education, and media-based education (10), are effective in patient acceptance. But new methods such as the use of technology, media (1), web applications (11), 3D online programs (12) and mobile applications (13, 14) may increase patient acceptance more, which is necessary to conduct more studies on the effectiveness of these methods (1). Reinforced Education is education with more emphasis and follow-up of preparation from the time of admission to the day of colonoscopy, which can be more effective with the use of media (15).

The level of satisfaction is an important criterion for evaluating the quality of services (16). Patient satisfaction is an indicator of quality in care and is known as an important factor in choosing a treatment center, so it is very important to conduct a study to find a way that improves the quality of services and patients satisfaction (17). It is necessary to conduct more studies in the field of improving the satisfaction of care recipients (18, 19). Incomplete preparation, misdiagnosis and repeated colonoscopies are the factors that reduce patient satisfaction, which can be resolved with proper training and appropriate follow-up. Based on the above information, it can be concluded that with the use of Reinforced Education by following the instructions, the bowel preparation and patient satisfaction can be improved, so this study was conducted with the aim of evaluating the effect of Reinforced Education on the satisfaction of the patients undergoing colonoscopy.

**Methods**

This research was semi-experimental with the aim of evaluating the effect of using media and cyberspace in educating colonoscopy patients referred to Ayatollah Taleghani Medical Educational Center. The statistical population of the research were included colonoscopy patients referred to Ayatollah Taleghani Educational Medical Center and the research sample was patients with inclusion criteria. Sampling was done on May 22_nd_ to June 22_nd_. (According to Iranian SH: Khordad 1_st_ to Tir 1_st_). The inclusion criteria were: not having movement, hearing and vision disorders, not having a medical history of suffering from cognitive diseases and depression, not taking neuroleptics, not having a new crisis such as the death of loved ones and hospitalization and finally access to cyberspace and educational videos. Exclusion criteria were patients who experienced a new crisis during the research or did not want to continue cooperation. The study was registered in the Iranian Clinical Trial Registry (IRCT20210131050189N5).

The number of samples is obtained using the following formula in each group:

$$n\geq2\frac{\left( z_{\alpha/2}+z_{\beta} \right)^{2} \sigma^{2}}{(\mu_{1}- \mu_{2})}$$

Probability of type one error: $\alpha=0.05 \to z_{\alpha/2}=1.96$

Probability of type two error:	$\beta=0.10 \to z_{\beta}=1.28$

Power of observed effect size: $\left( \mu_{1}- \mu_{2} \right)/{\sigma=0.75}$

Minimum required sample: $n=2\left( 1.96+1.28 \right)^{2} \left( \frac{1}{0.75} \right)^{2} =37$

At least 37 patients were required in each group. Considering a 40% population decline, the number of samples was included with 50 patients in each group.

The participants allocated into control and intervention groups by colored card (red and blue) (Figure 1). *RG* created the allocation sequence, *NF* and *SS* enrolled the participants, and authors *NF* and *RG* assigned the interventions to each group. *(Capital letters indicate the first names of the authors.)* First, the data of the control group was collected, and the patients in each group were blinded, meaning that they did not know whether they were assigned to the intervention group or the control group.

The homogeny of the two groups in the age, sex, education level, underlying disease, and the reason for admission was checked statistically and there were no significant differences.

Please insert figure 1 (CONSORT) here.

Bisacodyl tablets and Pidrolax powder were used for intestinal preparation. In the control group, the same previous training was given, which was given a printed training sheet, and in the intervention group, the patients were called the day before the colonoscopy, and training files including clips, application, and Instagram page were sent to them through WhatsApp software and their questions were answered.

The data collection tool included a demographic information questionnaire with 20 questions including the patient's age, sex, level of education, marital status, occupation, type of health insurance, reason for hospitalization, duration of hospitalization, history of illness, underlying disease, and medical family history.

The colonoscopy satisfaction instrument was a researcher-made instrument with 12 questions in 3 dimensions (pre-colonoscopy education, colonoscopy experience, and colonoscopy result). In order to prepare the tool, first the items of the tool were prepared based on the literature review and then, the items were categorized and summarized in the expert panel including 10 gastroenterologists and nursing experts and duplicate items were removed. In the next step, face validity (qualitative), content validity (quantitative and qualitative) and construct validity were examined. In order to check the validity of the content, 10 nurses were asked to provide their corrective comments in terms of following grammar, using appropriate words, placing expressions in their appropriate place and giving acceptable points. Then CVI and CVR were calculated and confirmed. In the next step, the relationship between the items was identified by exploratory factor analysis and summarized in 3 factors (pre-colonoscopy education, colonoscopy experience and colonoscopy result). Reliability was checked with Cronbach's alpha. Cronbach's alpha of the instrument was 0.83.

One week after the colonoscopy procedure, the patients were called and the aforementioned satisfaction questionnaire was completed.

Analyses were conducted using IBM SPSS Statistics, Version 20 (IBM Corp., Armonk, NY). The normality of distribution of variables was assessed by Kolmogorov-Smirnov tests. Statistically significance was presented as p value < 0.05. Demographic data analysis was done using either t test or chi-square test. If distribution of data was normal, paired t test and student t test were used to compare variables within and between groups, respectively.

it should be mentioned that we used the CONSORT reporting guidelines version 2010 for this manuscript (20).

**Results**

In this study 83 patients, including 47 men and 36 women, with an average age of 49.19 years old, participated. Table 1 shows their demographic characteristics in the intervention group (43 people) and the control group (40 people).

Please insert table 1 here

|  | | Group | | | |
| --- | --- | --- | --- | --- | --- |
|  |  | Control | | Intervention | |
|  |  | Count | N % | Count | N % |
| Gender | Female | 18 | 21.7% | 18 | 21.7% |
|  | Male | 25 | 30.1% | 22 | 26.5% |
| Marriage | Single | 7 | 8.6% | 6 | 7.4% |
|  | Marriage | 36 | 44.4% | 32 | 39.5% |
| Education | School | 17 | 21.5% | 15 | 19.0% |
|  | High School | 19 | 24.1% | 16 | 20.3% |
|  | Bachelor | 2 | 2.5% | 4 | 5.1% |
|  | Upper | 2 | 2.5% | 4 | 5.1% |
| Number colonoscopy | First | 29 | 34.9% | 19 | 22.9% |
|  | Second | 10 | 12.0% | 10 | 12.0% |
|  | More | 4 | 4.8% | 11 | 13.3% |
| Hospitalization | Outpatient | 33 | 39.8% | 35 | 42.2% |
|  | Inpatient | 10 | 12.0% | 5 | 6.0% |
| Family history | No | 33 | 40.2% | 29 | 35.4% |
|  | Yes | 10 | 12.2% | 10 | 12.2% |

The mean (standard deviation) of patient`s satisfaction in the intervention group was 11.78(4.65) and in the control group was 9.04 (2.95). The result of the independent t-test indicated a significant difference in the two groups (P< 0.01). In the colonoscopy experience factor, t-test showed that the difference is not significant, but in the other two factors patient`s satisfaction, colonoscopy results and pre-colonoscopy education, t-test showed the significant difference (P<0.05). Table 2 showed the results of patient satisfaction factors between control and intervention groups.

Please insert table 2 here

Table 2- Result of patient satisfaction factors between control and intervention groups

|  | Control | | Intervention | |  | |
| --- | --- | --- | --- | --- | --- | --- |
|  | Mean | Standard Deviation | Mean | Standard Deviation | t | P value |
| Pre-colonoscopy education | 1.66 | 1.68 | 3.55 | 2.51 | -3.99 | 0.00 |
| Colonoscopy experience | 4.57 | 2.17 | 4.95 | 2.13 | -0.68 | 0.49 |
| Colonoscopy result | 1.30 | 0.66 | 1.63 | 0.77 | -1.99 | 0.05 |

**Discussion**

This study was conducted with the aim of evaluating the effect of Reinforced Education on the level of satisfaction of the patients undergoing colonoscopy, and the results of the study indicated the effect of Reinforced Education with using media and cyberspace on improving the satisfaction of those patients (P<0.01). In this regard, Aggarwal et al have investigated the impact of training on safety and stated that effective training methods should be used regarding patient safety and emphasized on the continuation of training (21). In their study, Kristin A. Bott and colleagues investigated the effect of the implementation of a specialized training program after the treatment of cardiac arrhythmia in comparison with the standard discharge instructions, and in results of the study, they stated that the training provided to the patient by the nurse in 24 to 72 hour after discharge has improved patient satisfaction and reduced readmission rates (22). The results of the study by Christian A. Bot et al. are consistent with this study.

In this regard, Lutjeboer and colleagues have also stated according to the results of their study that promoting self-care can have an effect on patient satisfaction and can improve it. Their study was done in the radiology department (23). In the present study, training according to patients' needs had an effect on improving patients' satisfaction. Also, Ezzat et al. had similar results in their study, they have stated that reducing complications in cardiac catheterization can increase patient satisfaction (17). Since the reduction of complications is a part of self-care training, their results are also in line with the results of the present study and indicate an increase in satisfaction following the promotion of self-care with training and follow-up of care and preparations.

Patient education is essential to promote self-management and reduce hospitalization time and treatment costs (22). So that for every 1 dollar that is spent on educating the patient, 3 to 4 dollars are saved in costs. According to the statistics presented in the United States, about 69 to 100 million dollars are spent annually for medical problems caused by the lack of education (23).

Today, most people have mobile phones (24). Athilingam stated in 2016 that there are 7 billion mobile phones in the world and almost 95% of people in the world use mobile phones (25). The mobile phone is with its owner during the day (25) and its use is useful in teaching self-care and disease prevention programs (26) Convenient use and availability have   increased the satisfaction (27) Mobile is one of the most widely used technological advancements that improves the communication between the patient and the medical staff in the health system, and with suitable programs, higher attractiveness, and the possibility of remote patient follow-up, it has included satisfaction of the patients (28, 29). In the current study, mobile and media programs were used to improve education, and follow-up, and education with mobile in the reinforced educational program was more acceptable and increased patient satisfaction.

It is true that our study group was limited to patients undergoing colonoscopy, but it seems that the result can be generalized for other procedures where the background knowledge of the patient and his/her family is incomplete and limited, because it reduces the fear of the unknown in a new and understandable way.

**Research Limitations**

One of the limitations of the research was the outbreak of the Covid-19 disease, which interfered with data collection, but fortunately, using cyberspace and telephone follow-up of patients did not affect results. One of the strengths was the use of several teaching methods.

**Conclusions**

Based on the results, it can be concluded that Reinforced Education by using media and cyberspace is effective on the satisfaction of patients undergoing colonoscopy.

**List of abbreviation**

CVR: content validity ratio

CVI: content validity index

3D: 3 dimension

**Acknowledgment**

We would like to thank all participants and those who helped us in carrying out the research especially all the staffs of colonoscopy department of Ayatollah Taleghani Educational Medical Center.

**Authors’ contributions**

NF and RG conceived and designed the study.

NF, SS, and RG developed methodology.

NF, SS, and RG gathered, analyzed and interpreted the data.

NF and RG wrote and revised the manuscript.

RG supervised and coordinated all aspects of the work.

All authors have read and approved the manuscript.

**Funding**

We did not receive external funding for this research.

**Consent for publication**

Not applicable.

**Availability of Data and Materials:**

All data generated or analyzed during this study are included in this published article and its supplementary file

**Ethical Consideration**

The study protocol was approved with the Ethics Committee of Student Research Committee at Shahid Beheshti University of Medical Sciences (Ethics Approval No: IR.SBMU.RETECH.REC. 1400.1082) named *"Evaluation of the effect of reinforced education with using media on patient education on the satisfaction with colonoscopy"*. To observe the ethical considerations, the research goals and procedures were elucidated to the participants, they were assured of information anonymity and confidentiality, and informed written consent was obtained from each participants. They participated in the study voluntarily and could leave the study at any stage.

**Competing interests**

The authors declare that they have no competing interests.

**References**

1.	Kurlander JE, Sondhi AR, Waljee AK, Menees SB, Connell CM, Schoenfeld PS, et al. How efficacious are patient education interventions to improve bowel preparation for colonoscopy? A systematic review. PloS one. 2016;11(10):e0164442.

2.	Jeon SC, Kim JH, Kim SJ, Kwon HJ, Choi YJ, Jung K, et al. Effect of sending educational video clips via smartphone mobile messenger on bowel preparation before colonoscopy. Clinical endoscopy. 2019;52(1):53.

3.	Elvas L, Brito D, Areia M, Carvalho R, Alves S, Saraiva S, et al. Impact of personalised patient education on bowel preparation for colonoscopy: prospective randomised controlled trial. GE-Portuguese Journal of Gastroenterology. 2017;24(1):22-30.

4.	Sadeghi A, Rajabnia M, Bagheri M, Jamshidizadeh S, Saberi S, Shahnazi P, et al. Predictive factors of inadequate bowel preparation for elective colonoscopy. Gastroenterology and Hepatology From Bed to Bench. 2022;15(1):66.

5.	Schooley B, San Nicolas-Rocca T, Burkhard R. Cloud-based multi-media systems for patient education and adherence: a pilot study to explore patient compliance with colonoscopy procedure preparation. Health Systems. 2021;10(2):89-103.

6.	Marble N, Loescher LJ, Lim KH, Hiscox H. Use of technology for educating melanoma patients. Journal of Cancer Education. 2010;25(3):445-50.

7.	Cook DJ, Moradkhani A, Douglas KSV, Prinsen SK, Fischer EN, Schroeder DR. Patient education self-management during surgical recovery: combining mobile (iPad) and a content management system. Telemedicine and e-Health. 2014;20(4):312-7.

8.	Bernstein MT, Kong J, Sriranjan V, Reisdorf S, Restall G, Walker JR, et al. Evaluating information quality of revised patient education information on colonoscopy: It is new but is it improved? Interactive journal of medical research. 2019;8(1):e11938.

9.	Guo X, Li X, Wang Z, Zhai J, Liu Q, Ding K, et al. Reinforced education improves the quality of bowel preparation for colonoscopy: An updated meta-analysis of randomized controlled trials. PloS one. 2020;15(4):e0231888.

10.	Bernstein MT, Garber J, Faucher P, Reynolds KA, Restall G, Walker JR, et al. New Patient Education Video on Colonoscopy Preparation: Development and Evaluation Study. JMIR human factors. 2020;7(4):e15353.

11.	Trasolini R, Nap-Hill E, Suzuki M, Galorport C, Yonge J, Amar J, et al. Internet-Based Patient Education Prior to Colonoscopy: Prospective, Observational Study of a Single Center’s Implementation, with Objective Assessment of Bowel Preparation Quality and Patient Satisfaction. Journal of the Canadian Association of Gastroenterology. 2020;3(6):274-8.

12.	Zhao Y, Xie F, Bai X, Yang A, Wu D. Educational virtual reality videos in improving bowel preparation quality and satisfaction of outpatients undergoing colonoscopy: protocol of a randomised controlled trial. BMJ open. 2019;9(8):e029483.

13.	Walter B, Schmid R, von Delius S. A smartphone app for improvement of colonoscopy preparation (ColoprAPP): Development and feasibility study. JMIR mHealth and uHealth. 2017;5(9):e7703.

14.	Cho J, Lee S, Shin JA, Kim JH, Lee HS. The impact of patient education with a smartphone application on the quality of bowel preparation for screening colonoscopy. Clinical endoscopy. 2017;50(5):479.

15.	Walter B, Klare P, Strehle K, Aschenbeck J, Ludwig L, Dikopoulos N, et al. Improving the quality and acceptance of colonoscopy preparation by reinforced patient education with short message service: results from a randomized, multicenter study (PERICLES-II). Gastrointestinal endoscopy. 2019;89(3):506-13. e4.

16.	Ghazi K, Foroughan M, Hosseini M, Hosseinzadeh S, Askari M. The client satisfaction of delivered services in private nursing homes for elderly: a survey in the provinces of Golestan, Mazandaran, Semnan and Northern Khorasan in 2012 or 1391. Journal of Sabzevar University of Medical Sciences. 2013;20(3):320-30.

17.	Ezzat VA, Chew A, McCready JW, Lambiase PD, Chow AW, Lowe MD, et al. Catheter ablation of atrial fibrillation—patient satisfaction from a single-center UK experience. Journal of Interventional Cardiac Electrophysiology. 2013;37(3):291-303.

18.	Tevis SE, Kennedy GD, Kent KC. Is there a relationship between patient satisfaction and favorable surgical outcomes? Advances in surgery. 2015;49(1):221.

19.	Al-Abri R, Al-Balushi A. Patient satisfaction survey as a tool towards quality improvement. Oman medical journal. 2014;29(1):3.

20.	[Available from: <https://www.equator-network.org/reporting-guidelines/consort/>.

21.	Aggarwal R, Mytton OT, Derbrew M, Hananel D, Heydenburg M, Issenberg B, et al. Training and simulation for patient safety. BMJ Quality & Safety. 2010;19(Suppl 2):i34-i43.

22.	Bott KA. Effect of Enhanced Patient Education on Patient Satisfaction and 30-Day Readmission Rates after Cardiac Arrhythmia Ablation. 2016.

23.	Lutjeboer J, Burgmans MC, Chung K, van Erkel AR. Impact on Patient Safety and Satisfaction of Implementation of an Outpatient Clinic in Interventional Radiology (IPSIPOLI-Study): a quasi-experimental prospective study. Cardiovascular and interventional radiology. 2015;38(3):543-51.

24.	Cannon C, editor Telehealth, mobile applications, and wearable devices are expanding cancer care beyond walls. Seminars in oncology nursing; 2018: Elsevier.

25.	Athilingam P, Osorio RE, Kaplan H, Oliver D, O’neachtain T, Rogal PJ. Embedding patient education in mobile platform for patients with heart failure: theory-based development and beta testing. CIN: Computers, Informatics, Nursing. 2016;34(2):92-8.

26.	Kahn JG, Yang JS, Kahn JS. ‘Mobile’health needs and opportunities in developing countries. Health affairs. 2010;29(2):252-8.

27.	Thakkar J, Kurup R, Laba T-L, Santo K, Thiagalingam A, Rodgers A, et al. Mobile telephone text messaging for medication adherence in chronic disease: a meta-analysis. JAMA internal medicine. 2016;176(3):340-9.

28.	Skinner C, Finkelstein J, editors. Review of mobile phone use in preventive medicine and disease management. Proceedings of the IASTED International Conference on Telehealth/Assistive Technologies; 2008: ACTA Press.

29.	Liu C, Zhu Q, Holroyd KA, Seng EK. Status and trends of mobile-health applications for iOS devices: A developer's perspective. Journal of Systems and Software. 2011;84(11):2022-33.
