# Supplementary material for: Evaluation of the effect of reinforced education on the satisfaction of patients undergoing colonoscopy: A randomized controlled trial
Source: PLoS One. 2024 Jan 5;19(1):e0296126. doi: 10.1371/journal.pone.0296126 (PMC10769074; doi:10.1371/journal.pone.0296126)
Supplement: Attachment — Submitted filename: Response to Reviewers.docx [file pone.0296126.s004.docx]

Response to Reviewers

Dear Dr. Brillantino

Thank you for giving us the opportunity to submit a revised draft of our manuscript titled [Evaluation of the effect of reinforced education on the satisfaction of the patients undergoing colonoscopy: a randomized controlled trial] to *PLOS ONE*. We appreciate the time and effort that you and the reviewers have dedicated to providing your valuable feedback in our manuscript. We are grateful to the reviewers for their insightful comments on our paper. We have been able to incorporate changes to reflect most of the suggestions provided by the reviewers.We have highlighted the changes within the manuscript.

**Here is a point-by-point response to the reviewers` comments and concerns:**

| **Reviewer Comments** | **Response to The Reviewer** | **Page in Manuscript** |
| --- | --- | --- |
| **Journal Requirements:** | | |
| **1.** Ensure that your manuscript meets PLOS ONE's style requirements. | We checked the style approved by PLOS ONE following the two links sent in the email and reflected in our manuscript.  The changed parts are highlighted in the "track changes" file. | All of manuscript |
| **2-1.** your reasons for your delay in registering this study (after enrolment of participants started). | In fact, we did not know that obtaining the trial registration code is a necessity, so we settled for obtaining the code of ethics at first. After reading the guidelines and the recommendation of PLOS ONE, we registered our trial, which was successfully conducted and approved. (Documents previously attached for the journal)  For this reason, the registration of the trial was after obtaining the code of ethics and sampling procedures. | -- |
| **2-2.** confirmation that all related trials are registered by stating. | We added it. | 15 |
| **3.** State the name of the registry and the registration number in the submission data and on the title page of your manuscript. | We added it. | 1,5,15 |
| **3-a.** provide the complete date range for participant recruitment and follow-up in the methods section of your manuscript. | We added it. | 6 |
| **3-b.** confirm that all related trials are registered by stating: “The authors confirm that all ongoing and related trials for this drug/intervention are registered”. | We added it. | 5,15 |
| **4.** We note that the original protocol that you have uploaded as a Supporting Information file contains an institutional logo. As this logo is likely copyrighted, we ask that you please remove it from this file and upload an updated version upon resubmission. | The institutional logo of the original protocol was removed and uploaded again. | -- |
| **5.** Amend either the abstract on the online submission. | Sure.  Done. | -- |
| **6.** Include captions for your Supporting Information files at the end of your manuscript, and update any in-text citations to match accordingly. | We added it. | 20 |
| **Reviewer 1**: | | |
| The scientific writing of the article is not enough. | With the help of *Tarjomic* Institute, we wrote the article natively and uploaded its certificate to the journal as supporting information. | -- |
| File (by reviewer 1) | We are very grateful for your detailed and insightful comments, which greatly helped to improve the quality of our paper.  We have reviewed your comments one by one:   - If there was a typographical or spelling error, we corrected it. (Actually, we have not included this category of corrections below, but we have highlighted the corrections in the revised manuscript.) - If you have asked a question, we have answered it in the text of the manuscript to make it clearer for the reviewers and readers. - If the information provided by us was incomplete somewhere, we corrected and completed that part and matched it with your order or the article referred by you. - Incomplete references were corrected and matched. - Where our writing was ambiguous, we gave a clearer explanation.   All these items can be seen highlighted in the text of the article (track changes file). |  |
| Title: randomized controlled trial | Corrected. | 1,2 |
| Abstract: Results: p < 0.001 | Corrected. | 3 |
| The esge guideline recommendation should be appended with the level of evidence. | Our bowel preparation was in accordance with the ESGE guidelines, which were included in the text of the paper and added to the references. | 8 |
| What is the annual number of colonoscopies performed at Ayatollah Taleghani Medical Educational Center?  What is the average number of patients per month? | We added answers of these questions in the Methods Section.  "This medical center performs approximately 2,500 colonoscopies annually, or nearly 200 per month." | 5 |
| WHEN WAS THE DATA COLLECTED? | We answered this question completely in the Methods Section, data collection and sampling part.  "The sampling period lasted from May 22 to June 22. Data collection was completed through August 7. | 6 |
| How is the bowel preparation protocol routine?  What is the dose frequency and amount? How many days does it take? Is it just medical preparation?  No dietary restrictions?  When is oral intake stopped? | You are right and we did not explain about this. But now in the methods section, intervention part, we have fully described the routine protocol and its details. | 8-9 |
| When was the questionnaire administered and where was it administered? | We answered this question in the Methods Section, data collection part.  "Questionnaire questions were asked by the sampler to the sample people and entered into Google form at the same moment in the hospital." | 7 |
| Write down all the questions. 20 variables are not included here. | age, sex, level of education, marital status, occupation, type of health insurance, the reason for hospitalization, length of hospitalization, illness history, underlying disease, medical family history, previous colonoscopy experience, place of residence, diet (daily fruit consumption, weekly fast food consumption), ability to perform daily activities, and amount of exercise per day.consumption, weekly fast food consumption), ability to perform daily activities, amount of exercise per day. | 7 |
| Methods: Citations are added. | We added. | 7 |
| Methods: Endoscopy nurses or nurses? | endoscopy nursing experts | 7 |
| Table 2: Add the statistical significance criterion. If p < 0.05, this value (0.049) cannot be significant. | Yes, you`re right and 0.05 cannot be significant. | 11 |
| The studies used in the discussion cover different samples. Moreover, they did not receive training on bowel preparation. Does not match your goals (eg: length of stay etc.)  The discussion should be rewritten in relation to the findings. The sample should include colonoscopy patients, education bowel preparation instructions. Furthermore, the results should measure patient satisfaction. | Thanks for your consideration. Actually, these studies are presented in the discussion section due to their similarity in the topic of patient education and its effectiveness | 11-12 |
| I DID NOT UNDERSTAND THIS LIMITATION. BOTH PREPARATION INCLUDES ONLY PATIENTS WITH A COLONOSCOPY ALREADY. WHAT DO YOU MEAN? | We explained this part more clearly at the end of the discussion section. | 13 |
| WHEN WAS THE DATA COLLECTED?  DID YOU COLLECT DATA DURING THE PANDEMIC? | Yes, and it is mentioned in the text. | 13 |
| **Reviewer 2:** | | |
| **1. Methods:** | | |
| An orderly manner is suggested.  The authors are advised to create separate subsections for each of the possible topics (whichever necessary), and that way produce a very clear writeup. | We separated the method section into smaller components and completed the requirements according to the CONSORT 2010. | 5-9 |
| **a.** the randomization and allocation concealment should be made very clear (they are NOT the same thing); the trial staff recruiting patients should NOT have the randomization list. Randomization should be prepared by the trial statistician, and he/she would not participate in the recruiting | You are right, a researcher who don`t educated or collected data, recruiting patients. | -- |
| **b**. The manuscript does not provide details on the randomization procedure; any reasoning, why a block randomization was not used, which is often recommended to ensure a balance in sample size across groups | That was about education and blinded for researcher who collected data. | -- |
| **c.** The authors write in the Abstract: "In order to prevent the dissemination of information between the two groups, first the data of the control group was collected". This is not clear! You may easily attain information blockade between the 2 groups via blinding, and a proper randomization procedure. More details needed | We collected data in deferent days based on allocation. | -- |
| **d.** Sample size/power: A sample size/power statement is made available, but its recommended to place it as a separate subsection within the Methods section | We corrected in. | 6 |
| **e-1.** Alternative analysis methods under data non-Gaussianity is not mentioned, such as using well-known nonparametric tests | We added it. Initially, we examined the homogeneity of the two groups and observed no significant difference (P>0.05). | 11 |
| **e-2.** Several additional covariates (via the questions) were also obtained. Why a formal regression analysis was not conducted?  **e-3.** It's not clear if data was collected longitudinally; please provide details. If that is the case, mixed modeling should be used. | The covariate and mixed model analysis was done considering the intervention and matching of the two groups. Added assimilation in sampling and results. | -- |
| **e-4.** Overall write up has various inconsistencies; please fix incomplete sentences. For example. | We corrected them. | -- |
| **2. Results & Conclusions:** | | |
| **a.** The authors should check that any statement of significance should be followed by a p-value in the entire Results section. | We added it. | 9-11 |
| **b.** Conclusions should state that the current findings are ONLY based on the random samples derived from an Iranian population, and should allude to future studies with much larger sample sizes and collected at other geographical areas to confirm the effectiveness of the RE procedure. | We corrected it. | 13-14 |
