# Supplementary material for: Evaluation of the effect of reinforced education on the satisfaction of patients undergoing colonoscopy: A randomized controlled trial
Source: PLoS One. 2024 Jan 5;19(1):e0296126. doi: 10.1371/journal.pone.0296126 (PMC10769074; doi:10.1371/journal.pone.0296126)
Supplement: Attachment — Submitted filename: Reviewer Plo.docx [file pone.0296126.s005.docx]

8/16/2023

Editor-in Chief

PLOS ONE

Thank you very much for giving me an opportunity to review **Manuscript Number : PONE-D-23-02398R1 ,**entitled Evaluation of the effect of reinforced education on the satisfaction of

patients undergoing colonoscopy: a randomized controlled trial.”

This manuscript is suitable for publication in domestic and student educational Journal. Therefore, it cannot be published in PLOS ONE

With best wishes,

Dr.Durdi Qujeq

Faculty of Medicine , Babol University of Medical Sciences
