# Supplementary material for: Evaluation of the effect of reinforced education on the satisfaction of patients undergoing colonoscopy: A randomized controlled trial
Source: PLoS One. 2024 Jan 5;19(1):e0296126. doi: 10.1371/journal.pone.0296126 (PMC10769074; doi:10.1371/journal.pone.0296126)
Supplement: Attachment — Submitted filename: Response to Reviewers.docx [file pone.0296126.s007.docx]

Response to Reviewers

Dear Dr. Brillantino

Thank you for giving us the opportunity to submit a revised draft of our manuscript again. We hope that in this series of revised files, all the things you and your perfect reviewers want will be corrected and approved.

Many thanks.

**Here is a point-by-point response to the reviewers` comments and concerns:**

| **Reviewer Comments** | **Response to The Reviewer** | **Page in Manuscript** |
| --- | --- | --- |
| **Journal Requirements:** | | |
| Please review your reference list to ensure that it is complete and correct. | All articles were reviewed one by one. None of them were retracted. Did you mean this for a specific article? In this case, please provide the reference number so that I can check again. | 20-23 |
| **Reviewer Comments:** | | |
| **Reviewer 2:** | We are glad that we have answered your previous reviews and that you are satisfied.  In response to your question: T-test was used due to homogeneity.  In the *data analysis* section, statistical generalities and t-test have been discussed, and we think we have provided sufficient explanations.  Do you think it is advisable to add another explanation? | related to statistics |
| **Reviewer 4:** | Your attention to detail and the time you have spent on revising our manuscript are commendable. We appreciate your efforts. And thank you very much.  According to your order, the text has been edited natively and the certificate of the translation institute has been uploaded.  All the mentioned items were revised one by one and marked with a yellow highlight in the main text of the article in the *track change file*.  We hope it will be approved by you. | Whole pages. |
| **Reviewer 6:** | We are very pleased that the manuscript is acceptable to you in terms of the importance of the topic and the structure. Also, thank you for your reviews and taking the time.  Response: (point by point)   - I re-read the introduction several times. The body of this introduction has 3 main parts: In the first part, we have discussed about epidemiology and the importance of bowel preparation for colonoscopy. In the second paragraph, we mentioned the obstacles to proper preparation by patients and we mentioned the impact of training to tolerate this process. In the third and fourth paragraphs, using the literature review that you mentioned, we screened effective teaching methods with emphasis on new techniques. And in the final paragraph, due to the fact that we emphasized the measurement of patient satisfaction in the study, we expanded the reason for it and discussed the role of satisfaction on the performance requested from the patient. During the introduction, we have tried to avoid mentioning obvious and trivial things. However, if there is a specific sentence or part that you want to delete, please let me know so that I can revise it again. - Regarding randomization, we have used the method of red and blue cards, which was mentioned in the method, but a detailed and step-by-step explanation was added according to your request. - Regarding blinding, it should be noted that the patients and the person who collected the data after the intervention did not know about the group allocation. - The demographic information table was reorganized and the last column was added. - Our only limitation was the timing of the study with the covid-19 pandemic. But this incident did not have a significant impact on our research, maybe only the number of patient referrals was reduced due to the fear of getting infected in the hospital environment, which also did not have a direct effect on the study. It can even be said that the use of online and modern remote methods came to our team's mind with the start of the pandemic and became bold throughout the world of education and created a transformation.   The effectiveness of the study results, as mentioned in the previous literature, was the intervention with several educational methods that strengthened the positive and better results.   - Our intervention was necessary to clean the intestine and was in the patient's favor, and no additional intervention was given. Considering the nutrients that can be included in this diet and the short period of time, the clear liquid diet was not dangerous for the patient. No other complications were reported from Pidrolox powder, except for its taste, which was taught to be adjusted using its combination with fragrant citrus juice. - Informed consent was obtained and it was possible to withdraw from the study at any time during the study period, which is mentioned in the *ethical considerations* section of the manuscript text.   All the mentioned items were marked with blue highlight in the *track change file.*  If you need another explanation in another part of the study, let me know. | Whole pages. |
| **Dr. Durdi Qujeq:**  **(attached file)** | Dear Dr. Durdi Qujeq  This intervention is not unique to Iran, and the strategy of educating and following patients all over the world can be useful, as we have mentioned in the introduction, referring to articles, that the problem of not preparing the bowel properly before colonoscopy is a universal and common problem.  For this educational intervention, which was focused on the patient and to prevent them from coming back again, additional cost and patient harassment, we found the valuable PLOS ONE journal suitable for its publication.  If there is a question about research, I will be happy to answer. Thank you for taking the time to read the manuscript. | - |
| **attached file**  **(pdf)** | The demographic information table was modified.  The intervention of the control group was described in detail.  Items are highlighted in blue in the *track change file*. | 7,8 |
