# Supplementary material for: Evaluation of the effect of reinforced education on the satisfaction of patients undergoing colonoscopy: A randomized controlled trial
Source: PLoS One. 2024 Jan 5;19(1):e0296126. doi: 10.1371/journal.pone.0296126 (PMC10769074; doi:10.1371/journal.pone.0296126)
Supplement: Attachment — Submitted filename: Response to Reviewers.docx [file pone.0296126.s008.docx]

Response to Reviewers

Dear Dr. Brillantino

Thanks for taking the time to promote the authors and the journal.

I have corrected the references and **highlighted them in red** in the text.

You can see the full description in the table below.

It is hoped that this time the manuscript will be fully approved.

**Many Thanks.**

| **Reviewer Comments** | **Response to The Reviewer** | **Page in Manuscript** |
| --- | --- | --- |
| **Journal Requirements:** | | |
| Please review your reference list to ensure that it is complete and correct. | **Ref No.22:** Hasballah SM, Shaor OAE, Mohamed MA, Mohamed AK. Assess nurses' knowledge and attitude for patient safety in cardiac catheterization unit. Assiut Scientific Nursing Journal. 2019;7(19):151-9.  **This reference has been removed.**  *  **Ref No.25:** [Available from: <https://www.equator-network.org/reporting-guidelines/consort/>.  **This reference was revised and according to the notice of the EQUATOR website about the lack of access to the Consort website, the following site was referenced:**  **https://www.goodreports.org/reporting-checklists/consort/.**  *  **Ref No.33:** Skinner C, Finkelstein J, editors. Review of mobile phone use in preventive medicine and disease management. Proceedings of the IASTED International Conference on Telehealth/Assistive Technologies; 2008: ACTA Press.  **This reference has been removed.**  *  **Ref No.34:** Liu C, Zhu Q, Holroyd KA, Seng EK. Status and trends of mobile-health applications for iOS devices: A developer's perspective. Journal of Systems and Software. 2011;84(11):2022-33.  **This reference has been removed.** | 17-21 |
